# Genome-Wide Identification and Expression Analysis of the Mitogen-Activated Protein Kinase Gene Family in Cassava

**DOI:** 10.3389/fpls.2016.01294

**Published:** 2016-08-30

**Authors:** Yan Yan, Lianzhe Wang, Zehong Ding, Weiwei Tie, Xupo Ding, Changying Zeng, Yunxie Wei, Hongliang Zhao, Ming Peng, Wei Hu

**Affiliations:** ^1^Key Laboratory of Biology and Genetic Resources of Tropical Crops, Institute of Tropical Bioscience and Biotechnology, Chinese Academy of Tropical Agricultural SciencesHaikou, China; ^2^College of Life Science and Engineering, Henan University of Urban ConstructionPingdingshan, China; ^3^Hainan Products Quality Supervision & Testing InstituteHaikou, China

**Keywords:** abiotic stress, cassava, expression, genome-wide identification, mitogen-activated protein kinase (MAPK)

## Abstract

Mitogen-activated protein kinases (MAPKs) play central roles in plant developmental processes, hormone signaling transduction, and responses to abiotic stress. However, no data are currently available about the MAPK family in cassava, an important tropical crop. Herein, 21 *MeMAPK* genes were identified from cassava. Phylogenetic analysis indicated that MeMAPKs could be classified into four subfamilies. Gene structure analysis demonstrated that the number of introns in *MeMAPK* genes ranged from 1 to 10, suggesting large variation among cassava *MAPK* genes. Conserved motif analysis indicated that all MeMAPKs had typical protein kinase domains. Transcriptomic analysis suggested that *MeMAPK* genes showed differential expression patterns in distinct tissues and in response to drought stress between wild subspecies and cultivated varieties. Interaction networks and co-expression analyses revealed that crucial pathways controlled by MeMAPK networks may be involved in the differential response to drought stress in different accessions of cassava. Expression of nine selected *MAPK* genes showed that these genes could comprehensively respond to osmotic, salt, cold, oxidative stressors, and abscisic acid (ABA) signaling. These findings yield new insights into the transcriptional control of *MAPK* gene expression, provide an improved understanding of abiotic stress responses and signaling transduction in cassava, and lead to potential applications in the genetic improvement of cassava cultivars.

## Introduction

Mitogen-activated protein kinases (MAPKs), a specific class of serine/threonine protein kinases, are conserved throughout eukaryotes, including animals, yeasts, and plants (Hamel et al., 2006). There are multiple MAPK cascades existing in eukaryotic cells, which play a vital role in regulating gene expression, mitosis, metabolism, motility, survival, apoptosis, and differentiation (Marie and Roux, 2011). MAPK cascades are evolutionarily conserved signaling modules and are composed of at least three protein kinases: MAPKK kinases (MAPKKKs), MAPK kinases (MAPKKs) and MAPKs, which activate each other by phosphorylation in turn (Jonak et al., 2002). Activated MAPK cascade (MAPKKK → MAPKK → MAPK) kinases can regulate transcription factors and other components related to the MAPK pathway by phosphorylation. Based on the phylogenetic analysis of amino acid sequences and phosphorylation motifs, MAPKs in plants can be divided into four subfamilies (A, B, C, and D). Members of the A, B, and C subfamily have a Thr-Glu-Tyr (TEY) phosphorylation motif in their active sites, while members in the D subfamily have a Thr-Asp-Tyr (TDY) motif in their active sites and a long C terminal sequence (Ichimura et al., 2002).

Numerous studies have confirmed that *MAPK* family genes are involved in various abiotic stresses in plants, including drought, low temperature, high salt, osmotic stress, and hormone signaling. In rice, there are certain genes identified as positive regulators of abiotic stresses, such as *OsMPK2, OsMPK3, OsMAPK4, OsMAPK5*, and *OsMAPK44* (Fu et al., 2002; Huang et al., 2002; Xiong and Yang, 2003; Jeong et al., 2006; Xie et al., 2012). For example, overexpression of *OsMAPK5* and *OsMAPK44* separately in rice could greatly improve salt tolerance of transgenic plants (Xiong and Yang, 2003; Jeong et al., 2006). Nonetheless, *OsMAPK33*-overexpressing lines exhibited a higher sensitivity to salt stress than the wild type (Lee et al., 2011). In Arabidopsis, some *MAPK* genes are also involved in abiotic stresses, such as *AtMPK1, AtMPK2, AtMPK3, AtMPK4, AtMPK6, AtMPK7, AtMPK9, AtMPK11, AtMPK12, AtMPK17*, and *AtMPK18* (Ichimura et al., 2000; Teige et al., 2004; Dóczi et al., 2007; Ortiz-Masia et al., 2007; Khaled et al., 2008; Menges et al., 2008; Xing et al., 2008; Jammes et al., 2011). For instance, *AtMPK9* and *AtMPK12* are positive regulators of reactive oxygen species (ROS)-mediated ABA signaling in guard cells (Jammes et al., 2009). Loss of function of *MPK6* in Arabidopsis enhances cadmium tolerance by alleviating oxidative injury (Jin et al., 2013). However, *AtMPK4* functions as a negative regulator under osmotic stress (Droillard et al., 2004). These studies illustrate that MAPKs are positively or negatively involved in abiotic stress response, indicating that *MAPK*-mediated abiotic stress response is complex in plants.

So far, many members of the *MAPK* family have been identified using functional genomic methods. Twenty *MAPKs* have been found in Arabidopsis, 15 in rice, and 19 in maize (Ichimura et al., 2002; Rohila and Yang, 2007; Liu et al., 2013). Besides, *MAPKs* in many species, such as *B. distachyon*, banana, apple, tomato, and cucumber, have also been identified (Chen et al., 2012; Kong et al., 2012; Zhang et al., 2013; Asif et al., 2014; Wang et al., 2015). However, no genome-wide information has been available for the *MAPK* gene family in cassava. Cassava (*Manihot esculenta* Crantz) is one of the top three important root and tuber crops in the world (Hu et al., 2015a). Due to its effective utilization of light, heat, and water resources, it has high tolerance to drought and sterile soil, as well as a high starch accumulation in storage roots (Hu et al., 2015b); thus it is also used as a major raw material for non-grain based biofuels in China (Tawanda et al., 2012; Perera et al., 2014). With the aid of genomics tools, the fundamental research field of cassava has been focused on starch storage root development, starch accumulation, and stress response and regulation (Wang et al., 2014; Zeng et al., 2014). However, little information is known about the mechanisms of cassava responding to abiotic stress. Thus, understanding of the molecular mechanisms underlying cassava tolerant to abiotic stress will provide effective ways for genetic improvement of stress tolerance for cassava and other crops. The recently completed genome sequencing project for a wild ancestor and a domesticated variety of cassava provides an excellent opportunity for genome-wide analysis (Wang et al., 2014). Due to the importance of *MAPKs* in diverse biological and physiological processes as well as their potential application for the development of improved stress-tolerant transgenic plants, we performed a systematic analysis of the *MAPK* family in cassava. Based on the complete genome sequence and transcriptomic data, we identified 21 *MAPK* genes from the cassava genome, and investigated their phylogeny, conserved motifs, gene structure, and interaction networks, as well as expression profiles in various tissues and in response to drought. Furthermore, the expression profiles of *MeMAPK* genes in response to osmotic, salt, cold, ABA, and H_2_O_2_ were analyzed using quantitative real-time polymerase chain reaction (qRT-PCR). The identification and comprehensive investigation of the *MAPK* gene family in cassava will provide useful information for future research on the function of *MAPK* gene family and genetic improvement of cassava resistance to abiotic stress.

## Materials and methods

### Plant materials and treatments

The South China 124 (SC124) is a widely cultivated cassava variety in China (Zeng et al., 2014). The Argentina 7 (Arg7) is a kind of high starch content of cassava varieties (Zhao et al., 2014). W14 (*Manihot esculenta* ssp. *flabellifolia*) is an ancestor of the wild cassava subspecies with a strong drought resistance (Wang et al., 2014). All the materials were planted in a glass house in Chinese Academy of Tropical Agricultural Sciences (Haikou, China) during April to July 2013, because the temperature ranged from 20 to 35°C in that time which is suitable for cassava growing.

Stems (90 days after planting), leaves (90 days after planting) and storage roots (150 days after planting) were collected from Arg7 and W14 under normal growth conditions. Each sample contains 10 stems/leaves/storage roots from the same position of independent plants. These samples were subjected to RNA extraction and subsequent RNA-seq analysis to study the transcriptional accumulation of *MAPK* genes in different tissues.

Ninety-days-old cassava seedlings similar in growth state were withheld water for 12 days, then the leaves and roots were collected from Arg7, SC124, and W14 under normal conditions and 12 days drought treatment, respectively. Each sample contains 10 leaves/roots (3 cm from the tips of leaves or roots) from the same position of independent plants. These samples were used to examine the transcriptional response of *MAPK* genes in response to drought stress by RNA-seq technique.

Sixty-days-old cassava seedlings with consistent growth state were subjected to various abiotic stress treatments, including 200 mM mannitol for 2, 6 h, 3, and 14 days, 300 mM NaCl for 2, 6 h, 3, and 14 days, 10% H_2_O_2_ for 2, 6, 10, and 24 h, 100 μM ABA for 2, 6, 10, and 24 h, and cold (4°C) for 2, 5, 15, and 48 h, respectively. Each treated sample contained a corresponding regularly-watered control and each sample had three independent biological replications. Then, the treated and control plants at each time point were sampled to detect the expression patterns of *MeMAPK* genes by qRT-PCR.

### Identification and phylogenetic analyses of the MAPK family in cassava

The whole protein sequence of cassava was downloaded from the cassava genome database (http://www.phytozome.net/cassava.php). The Arabidopsis MAPK amino acid sequences were downloaded from UniPort (http://www.uniprot.org/). The rice MAPK amino acid sequences were obtained from RGAP databases (http://rice.plantbiology.msu.edu/). The local Hidden Markov Model-based searches (HMMER: http://hmmer.janelia.org/) built from all the known MAPK protein sequences from Arabidopsis and rice were used to identify the *MAPK* genes in cassava (Finn et al., 2011). Furthermore, BLAST analyses were employed to characterize the possible MAPKs with all the MAPKs from Arabidopsis and rice as queries. The predicted cassava *MAPK* genes were accepted only if they contained the MAPK domain validated by CDD (http://www.ncbi.nlm.nih.gov/cdd/) and PFAM databases (http://pfam.sanger.ac.uk/), and then the conserved domains of predicted MAPK sequences were confirmed by multiple sequence alignments. Further, the cassava MAPK sequences were reciprocally searched against the Arabidopsis and rice database to identify the best hit among all the *MAPK* genes. The proteins that did not contain the known conserved domains or the best hits of reciprocally searches were removed manually. Based on the multiple alignments of MAPKs from cassava, Arabidopsis, rice, tomato, apple, and cucumber, a bootstrap neighbor-joining (NJ) phylogenetic tree was constructed by Clustal X 2.0 and MEGA 5.0 (Larkin et al., 2007; Tamura et al., 2011). Tree reliability was assessed using 1000 bootstrap replicates.

### Protein properties and sequence analyses

With the help of the online ExPASy proteomics server database (http://expasy.org/), the molecular weight and isoelectric points of predicted MeMAPK proteins were speculated. The MEME program (http://meme-suite.org/) was applied to identify the conserved motifs of MeMAPKs. The maximum number of motifs was 12 and the optimum width of motifs was set from 15 to 50 [51]. The identified motifs were further annotated based on InterProScan (http://www.ebi.ac.uk/Tools/pfa/iprscan/). The gene structures of cassava *MAPKs* were analyzed with GSDS software (http://gsds.cbi.pku.edu.cn/) by comparison of coding sequence and genomic DNA sequence of each *MAPK* gene. Specific interaction network with experimental evidences of MAPK3, −4 and −6 were constructed using STRING (http://string-db.org/) with option value >0.97, which identifies 30 high confidence interactive proteins in Arabidopsis. Then, the homologs of these interactive proteins in cassava were identified with reciprocal best BLASTP analysis and their expression patterns after drought treatment were retrieved from RNA-seq data sets.

### RNA-Seq analysis

Total RNA was extracted using the plant RNeasy extraction kit (TIANGEN, China) with DNase I. First-strand complementary DNA (cDNA) was synthesized from total RNA by the RevertAid First Strand cDNA Synthesis Kit (Fermentas, USA). Second-strand cDNA synthesis was performed using DNA polymerase I and RNase H, and the cDNA fragments were processed for end repair, a single “A” base was added, and sequences were ligated to the adapters. The cDNA libraries were constructed following the Illumina protocols, which were sent to sequencing by Illumina GAII. A total of 610.70 million 51-bp raw reads was generated from the 18 samples. Adapter sequences were removed from raw sequence reads using FASTX-toolkit (version 0.0.13, http://hannonlab.cshl.edu/fastx_toolkit/). Sequence quality was examined using FastQC (http://www.bioinformatics.babraham.ac.uk/projects/fastqc/) and low quality sequences (including reads with unknown base pairs “N”) were removed, which produced 583.82 million clean reads. Clean reads were mapped to cassava reference genome (version 4.1) derived from the phytozome website (ftp://ftp.jgi-psf.org/pub/compgen/phytozome/v9.0/Mesculenta/) using Tophat v.2.0.10 (http://tophat.cbcb.umd.edu/) (Trapnell et al., 2009), and 88.7% reads were aligned. The resulting alignment files are provided as input for Cufflinks to generate transcriptome assemblies (Trapnell et al., 2012). Gene expression levels were calculated as FPKM according to the length of the gene and reads count mapped to this gene: FPKM = total exon fragments / [mapped reads (millions) × exon length (kb)]. DEGseq was applied to identify differentially expressed genes with a random sampling model based on the read count for each gene (Wang et al., 2010).

### qRT-PCR analysis

In order to detect the transcriptional levels of *MeMAPK* genes under various treatments (osmotic, salt, cold, ABA, and H_2_O_2_) and validate transcriptomic data, qRT-PCR were carried out with Stratagene Mx3000P Real-Time PCR system (Stratagene, CA, USA) using SYBR® Premix Ex Taq™ (TaKaRa, Japan) according to the manufacturer's instructions. Prior to quantification experiments, a series of primer and template dilutions were conducted to measure the optimal primer and template concentrations. Primers with high specificity and efficiency determined by melting curve analysis and agarose gel electrophoresis were selected to perform quantification assay. Moreover, PCR products were sequenced to confirm the specificity of primer pairs. The chosen primer pairs were listed in Table S1. Amplification efficiencies of primer pairs were between 0.93 to 1.06. Cassava *TUB* and *EF1* verified to be constitutive expression and suitable as internal controls was selected as reference genes to normalize the relative expression of target genes (Salcedo et al., 2014). The 2^−ΔΔCt^ method was used to calculate the relative expression level of the target genes (Livak and Schmittgen, 2001).

## Results and discussion

### Identification and phylogenetic analysis of cassava MAPKs

To identify *MAPK* family genes from the cassava genome, both BLAST and Hidden Markov Model searches were performed using Arabidopsis and rice MAPK proteins as query sequences. A total of 21 *MAPK* genes, designated as *MeMAPK1*-*MeMAPK21*, were obtained from the cassava genome, which were further supported by conserved domain and multiple sequence alignment analyses (Figure S1, Hamel et al., 2006). The 21 identified MeMAPK proteins from cassava ranged from 278 (MeMAPK20) to 602 (MeMAPK5) amino acids in length, with a relative molecular mass from 32.37 kDa (MeMAPK20) to 68.52 kDa (MeMAPK21) and protein pIs from 4.93 (MeMAPK20) to 9.20 (MeMAPK13 and MeMAPK21) (Table S2). We have submitted all of the cDNA sequences of these 21 MeMAPK genes to GenBank, and the corresponding accession numbers in GenBank are shown in Table S3.

**Figure 1:**
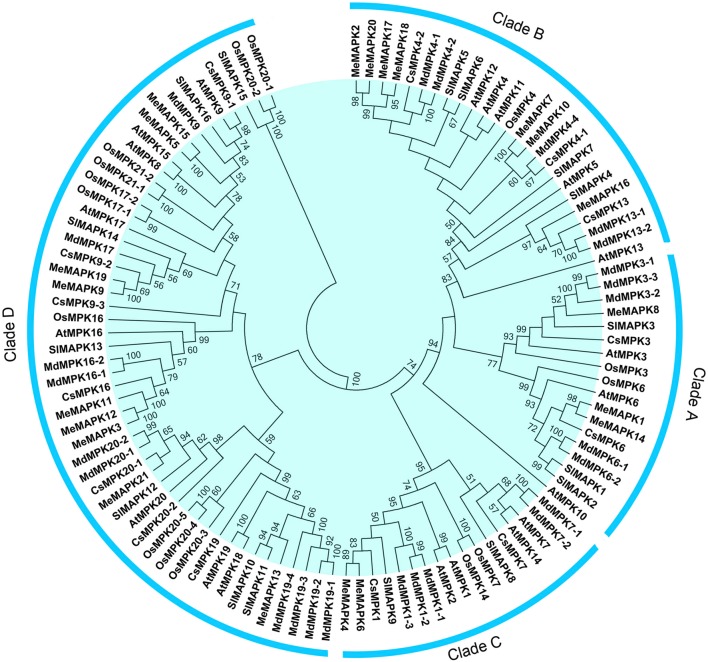
**Phylogenetic analysis of MAPKs in various species**. A total of 21 MeMAPKs from cassava, 20 AtMPKs from Arabidopsis, and 15 OsMPKs from rice, 16 SlMAPKs from tomato, 26 MdMPKs from apple, and 14 CsMPKs from cucumber, were used to create the NJ tree using ClustalX 2.0 and MEGA5 with 1000 bootstrap. Branches with less than 50% bootstrap support were collapsed. Four clades were labeled as A, B, C, and D.

In order to evaluate the evolutionary relationships among the MeMAPK proteins, an unrooted Neighbor-Joining phylogenetic tree was created with amino acid sequences of 21 MeMAPKs from cassava, 20 AtMPKs from Arabidopsis, 15 OsMPKs from rice, 16 SlMAPKs from tomato, 26 MdMPKs from apple, and 14 CsMPKs from cucumber (Table S3). As shown in Figure 1, 21 MeMAPKs were classified into four different groups (groups A, B, C, and D) together with their MAPK orthologs in various species, which is in accordance with previous phylogenetic classifications of MAPKs (Ichimura et al., 2002; Wang et al., 2015). Group A contained 3 genes, group B contained 7 genes, and group C contained 2 genes, while group D was the largest clade with 9 *MeMAPK* genes. Group D was also reported to be the largest subfamily of MAPK family in Arabidopsis, rice, poplar, and tomato (Ichimura et al., 2002; Nicole et al., 2006; Rohila and Yang, 2007; Kong et al., 2012). The classification of MAPKs suggests that *MeMAPKs* in different subgroups might have different functions.

According to the phylogenetic tree, many *MAPK* genes in cassava had paralogs among themselves, such as *MeMAPK2* and *MeMAPK20, MeMAPK17* and *MeMAPK18, MeMAPK7* and *MeMAPK10*. Besides, there were some closely related orthologous *MAPKs* between cassava and other dicotyledons, such as *MeMAPK7/10* and*MdMPK4-4/CsMPK4-1, MeMAPK8* and *MdMPK3-1/3-2/3-3, MeMAPK1/14* and *CsMPK6, MeMAPK4/6* and *CsMPK1, MeMAPK13* and *SlMAPK10/11*, MeMAPK21 and CsMPK20-1, MeMAPK3/11/12 and CsMPK16, MeMAPK9/19 and CsMPK9-2, while no orthologous *MAPKs* were found between cassava and rice. This indicated that *MAPKs* from cassava generally had a closer relationship with the proteins from dicotyledons than those from rice, which is accord with the current understanding of plant evolutionary history.

### Gene structure and conserved motifs of cassava MAPKs

**Figure 2:**
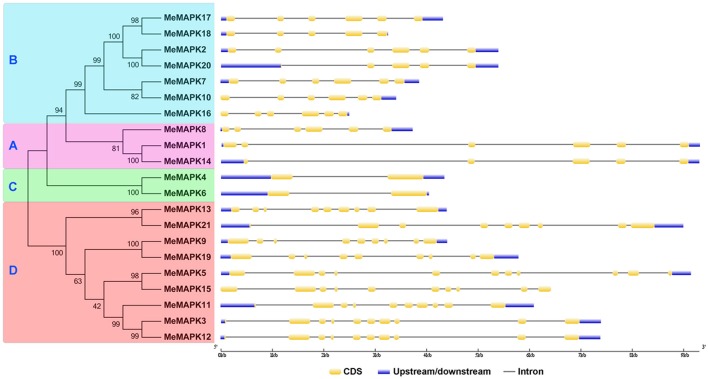
**The phylogenetic relationship and exon-intron structure analyses of MAPK family in cassava**. Exon-intron structure analysis was carried with online tool GSDS. Lengths of exons and introns of each *MeMAPK* gene were exhibited proportionally. A, B, C, and D indicated the classification of cassava MAPKs according to the phylogenetic relationship.

Gene structure divergence is important in the evolution of gene families and provides valuable evidence to assess phylogenetic relationships (Wang et al., 2015). The identification of exon/intron structures for each *MeMAPK* gene was studied by aligning full-length cDNA sequences and corresponding genomic DNA sequences (Figure 2). Groups A and B shared the same number of introns varying from 4 to 5, while the corresponding number in poplar and tomato were both 5 (Nicole et al., 2006; Kong et al., 2012). Group C only had 1 intron with a strictly conserved size, similar with the group C *MAPKs* in Arabidopsis, poplar, tomato, and apple (Ichimura et al., 2002; Nicole et al., 2006; Kong et al., 2012; Zhang et al., 2013). Group D had a larger number of introns with variable intron lengths than other groups. In this group, the number of introns varied from 8 to 10, which was much similar to other plants, including Arabidopsis, poplar, tomato, and apple (Ichimura et al., 2002; Nicole et al., 2006; Kong et al., 2012; Zhang et al., 2013). The speed of intron gain is much slower than the rate of intron loss after segmental duplication in rice (Nuruzzaman et al., 2010). It is thus possible that the group D may represent the original genes of MAPKs. Additionally, most members within the same group share a similar exon/intron structure, which further supports the classification of the *MeMAPK* genes in this study.

**Figure 3:**
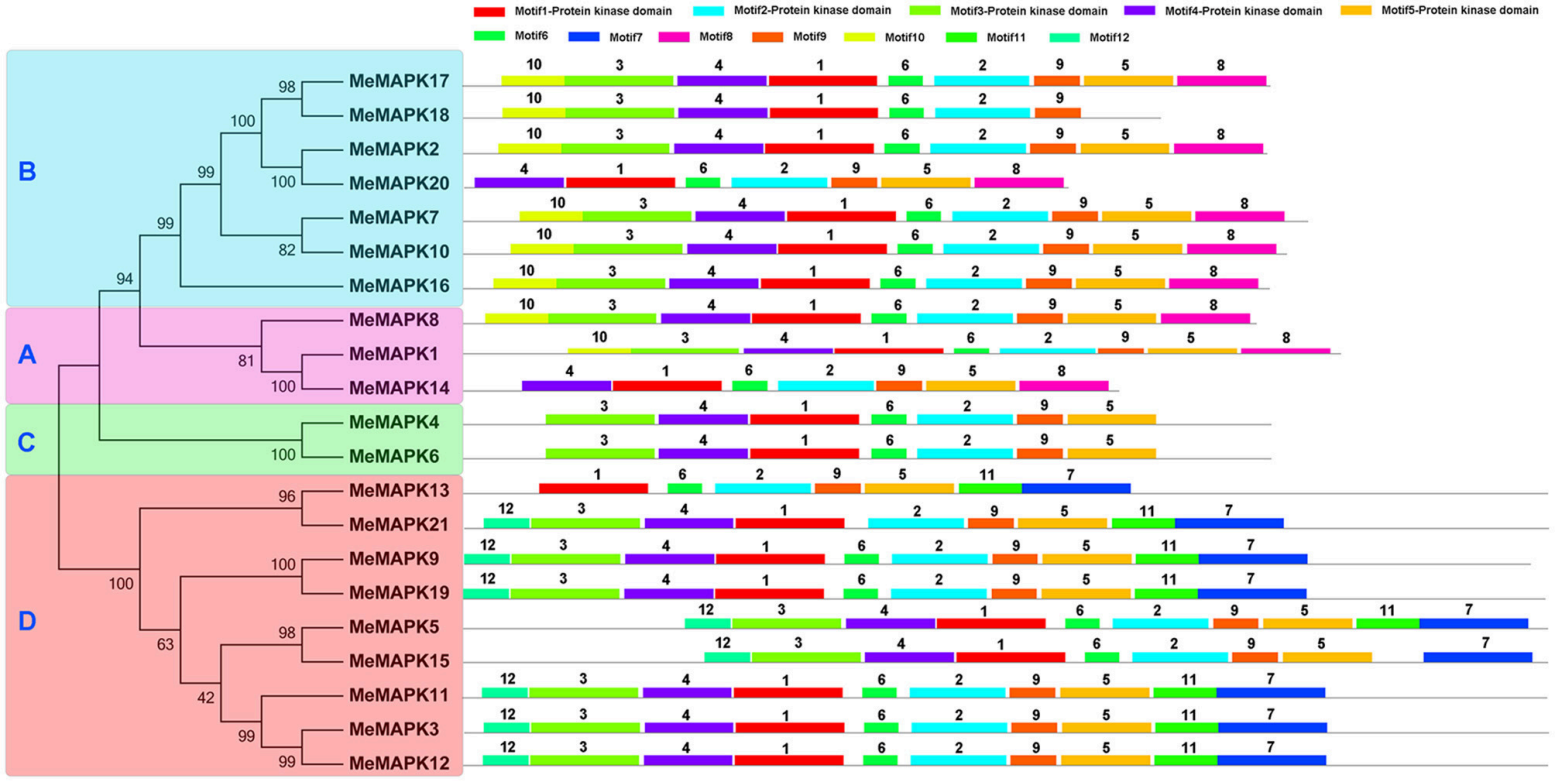
**Conserved motifs of cassava MAPKs according to the phylogenetic relationship**. All motifs were identified by MEME with the complete amino acid sequences of the 21 MAPKs from cassava. Lengths of motifs of each MeMAPK protein were displayed proportionally. A, B, C, and D indicated the classification of cassava MAPKs according to the phylogenetic relationship.

The MEME program was used to identify the conserved motifs of cassava MAPKs to explore structural diversity, and each motif was subsequently annotated with InterPro (Figure 3; Figure S3). As shown in Figure 3, 12 conserved motifs was identified, among which motifs 1–5 were annotated as the protein kinase domain. All of the identified MeMAPKs contained motifs 1 and 2, indicating that all of the cassava MAPKs were typical of the MAPK family. Additionally, the majority of MeMAPKs contained the 5 protein kinase motifs (motifs 1, 2, 3, 4, and 5), with the exception of MeMAPK13, MeMAPK14, MeMAPK18, and MeMAPK20. Specifically, group A contained 7 conserved motifs (motifs 1, 2, 4, 5, 6, 8, and 9), group B contained 5 conserved motifs (motifs 1, 2, 4, 6, and 9), and group D contained 5 conserved motifs (motifs 1, 2, 5, 7, and 9). The two members in group C contained completely identical motifs (motifs 1, 2, 3, 4, 5, 6, and 9), which was in agreement with the exon-intron structure of group C. In addition, along with all of the conserved motifs, most MAPK proteins in group A and group B had specific motif 10 at the N-terminal region and motif 8 at the C-terminal region, whereas most MAPKs in group D contained specific motif 12 at the N-terminal region as well as motifs 7 and 11 at the C-terminal region. This suggested functional consistency among the members in the same group. Moreover, motifs in group D were the most diverse, which was in accordance to the intron/exon structure of this group. Thus, the sequential order and the composition of these motifs in the same group showed a high similarity.

In addition, all of the MeMAPKs in groups A, B, and C contain the TEY motif at the phosphorylation site, whereas the members of group D, except for MeMAPK21, contain a TDY motif (Figure S2), which is consistent with previous findings from other species, such as Arabidopsis and rice (Hamel et al., 2006). Moreover, those MeMAPKs in group D have an extended C-terminal region compared to the other three groups (Figure S1), which is also present in Arabidopsis and *B. distachyon* (Ichimura et al., 2002; Chen et al., 2012).

### Expression patterns of cassava *MAPK* genes in different tissues

Accumulated evidences have confirmed that MAPKs play crucial roles in plant development. To better understand the function of *MeMAPK* genes in growth and development of cassava, transcription levels of *MeMAPK* genes in different tissues, including leaves, stems and the storage roots were examined in cultivated variety Arg7 and wild subspecies W14. According to the transcriptomic dataset, we found that six *MeMAPK* genes, including MeMAPK6, −*8*, −*12*, −*14*, −*18*, and −*20*, were not expressed. Interestingly, five of them were clustered with another MeMAPK gene into a sub-group (e.g., MeMAPK6/4, MeMAPK12/3, MeMAPK14/1, MeMAPK18/17, and MeMAPK20/2), respectively, based on our phylogenetic tree (Figure 1). The results indicated that these MeMAPK genes were probably generated through duplication events and one of them was silenced during the evolutionary process. A heat-map with hierarchical clustering was generated to display the expression patterns of 15 *MeMAPK* genes in different tissues (Figure S4; Table S4). Fourteen of 15 *MeMAPK* genes, except for *MeMAPK1*, were expressed in all the tested tissues of the two accessions. The transcripts of *MeMAPK1* were only detected in stems and leaves of W14, and storage roots of Arg7. In total, the number of *MeMAPK* genes with high expression levels (value >15.0) in stems, leaves, and storage roots of Arg7 were 7 (47%), 8 (53%), and 9 (60%), respectively, and the corresponding number in W14 were 6 (40%), 8 (53%), 6 (40%), respectively. In contrast, the number of *MeMAPK* genes with low expression levels (value < 5.0) in stems, leaves, and storage roots of Arg7 were 4 (27%), 3 (20%), 3 (20%), respectively, while 6 (40%), 2 (13%), and 4 (27%) were found in W14, respectively.

In addition, *MeMAPK5*, −*9*, −*11*, −*13*, and −*19* showed high expression levels (value >15.0) in stems of both Arg7 and W14. *MeMAPK2*, −*3*, −*5*, −*11*, −*13*, −*17*, and −*19* were predominantly expressed in the leaves of both Arg7 and W14. Similarly, *MeMAPK2*, −*5*, −*9*, −*11*, −*13*, and −*17* were highly expressed in storage roots of the two varieties. Therefore, these genes might play an important role in tissue development of cassava. In contrast, *MeMAPK1*, −*7*, −*10*, and −*16* had low expression levels (value < 5.0) in stems of both Arg7 and W14, *MeMAPK16* showed low expression level (value < 5) in the leaves of both Arg7 and W14, and *MeMAPK1*, −*7*, and −*10* exhibited low expression levels (value < 5) in storage roots of the two varieties. It is worth noting that 3 *MAPK* genes (*MeMAPK5*, −*11*, and −*13*) belonging to group D had high expression levels (value >15.0) in all of the tissues of both Arg7 and W14, indicating that these genes may function significantly in the growth and development stage in cassava (Kong et al., 2012).

### Expression analysis of cassava *MAPK* genes in response to drought in different varieties

**Figure 4:**
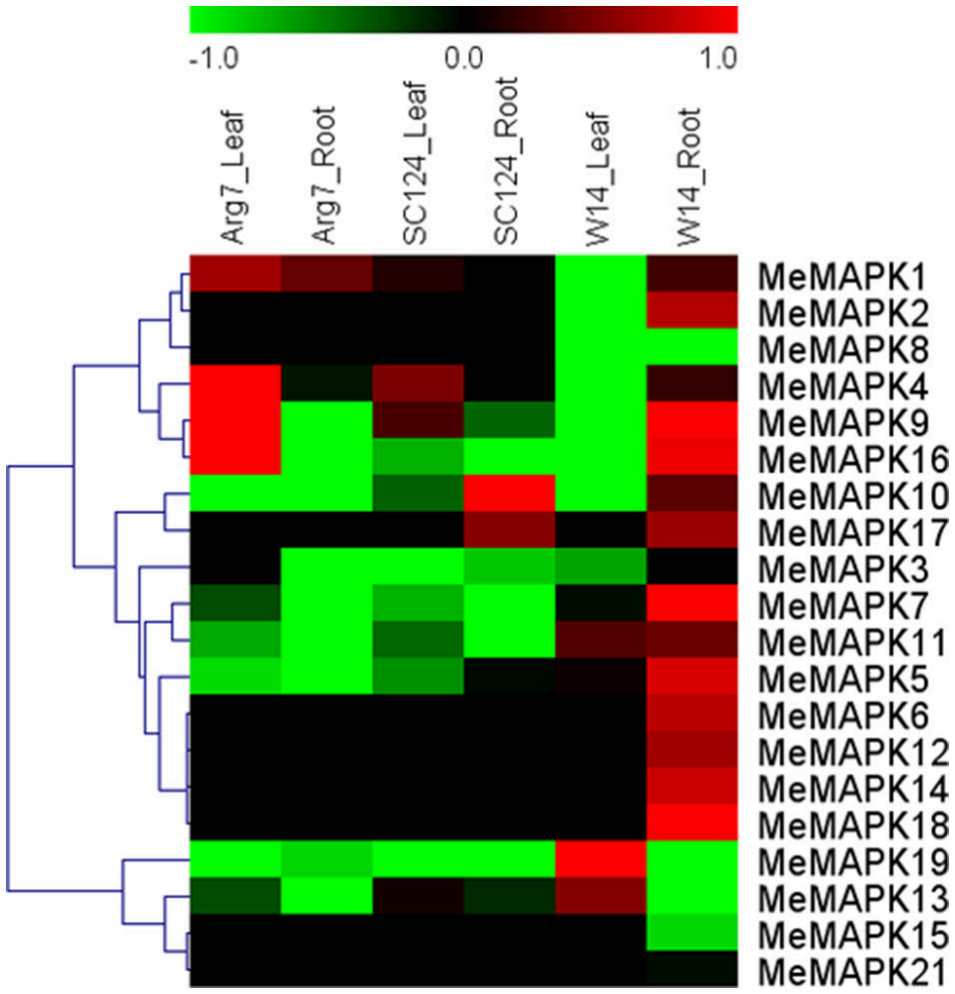
**Expression profiles of ***MAPK*** genes in leaves and roots of wild subspecies (W14) and two cultivated varieties (Arg7 and SC124) after drought treatment**. Log2 based FPKM value was used to create the heat map with clustering. The scale represents the relative signal intensity of FPKM values.

To gain insight into expression patterns and putative functions of *MeMAPKs* in response to drought stress, 3-month-old seedlings of W14, Arg7, and SC124 were subjected to drought treatment for 12 days. Then we extracted RNA from leaves and roots of the three varieties separately to complete subsequent RNA-seq analysis. The dataset had covered the corresponding transcripts data of all the *MeMAPK* genes except for *MeMAPK20* (Figure 4; Table S5). Based on the transcriptomic data, we found that 20% and 5% of *MeMAPK* genes in Arg7 were up-regulated by drought stress in leaves and roots, respectively. Similiarly, 20 and 10% of *MeMAPK* genes were induced by drought in leaves and roots of SC124, respectively. In the W14 subspecies, 20% of *MeMAPK* genes in leaves, and 70% in roots were found to be induced by drought stress. Thus, the total number of drought-induced *MeMAPK*s was much greater in W14 than that in Arg7 and SC124. As an ancestor of the wild cassava subspecies, W14 had greater drought tolerance than SC124 and Arg7 (Wang et al., 2014). Numerous evidences have confirmed that *MAPK* cascades play a positive role in response to drought or osmotic stress in plants (Ichimura et al., 2000; Teige et al., 2004; Kim et al., 2011; Li et al., 2013); therefore, we speculated that the high ratio of *MAPK* genes induced by drought in W14 might contribute to its strong drought tolerance. The response of plants to drought stress are complicated process, including perception of stress signals and their transduction that activate various stress-related genes and synthesis of proteins with diverse functions resulting in physiological and metabolic responses (Hu et al., 2013). Because there are some differences for genetic background between wild species (W14) and cultivated varieties (Arg7 and SC124, Wang et al., 2014), it is possible that a large number of genes showed differential expression patterns in response to drought stress between wild species and cultivated varieties. Thus, the contribution of MAPK genes to drought tolerance of cassava is to elucidate.

Additionally, based on transcriptomic data, the number of *MAPK* genes up-regulated by drought was greater in leaves than those in roots in Arg7 and SC124. On the contrary, the number of up-regulated *MAPK* genes was significantly more in roots than those in leaves in W14. Cassava can form deep root systems (below 2 m of soil depth), which is beneficial for penetrating into deeper soil layers and absorbing water stored in the soil (Okogbenin et al., 2013). Therefore, the high ratio of drought induced MeMAPK genes in roots indicates that MAPK genes may play a regulatory role in water uptake from soil by roots, hence maintaining strong tolerance to drought stress in W14. Due to the differences of genetic background between wild species (W14) and cultivated varieties (Arg7 and SC124), it is necessary to further confirm this hypothesis in future work.

Moreover, 3 genes (*MeMAPK5*, −*11*, −*19*) showed up-regulation in leaves of W14, whereas they were down-regulated in leaves of Arg7 and SC124. Additionally, 6 genes (*MeMAPK4*, −*5*, −*7*, −*9*, −*11*, and −*16*) were up-regulated in roots of W14, while they were down-regulated in roots of Arg7 and SC124. *MeMAPK* genes presented different expression patterns in different accessions, indicating that the roles of *MeMAPK* genes in drought stress response were different between wild subspecies and cultivated varieties. Some paralogous *MeMAPK* genes showed different transcriptional levels in response to drought, such as *MeMAPK1*, −*14* in group A, *MeMAPK4*, −*6* in group C, *MeMAPK3*, −*12* in group D, and *MeMAPK5*, −*15* in group D. Thus, in conclusion, the diversity of *MeMAPK* gene expression patterns under drought stress in wild subspecies and cultivated varieties might provide a direction for further research on the high drought tolerance mechanisms in cassava.

### The MAPK family interaction network and their co-expression in response to drought

To date, increasing evidence has confirmed that MAPK cascades are involved in a wide range of stress responses as a result of the interaction between MAPK proteins and various proteins, among which MPK3, MPK4, and MPK6 in Arabidopsis have been most extensively studied (Ichimura et al., 2000; Droillard et al., 2002; Jonak et al., 2002; Pitzschke et al., 2009). In recent years, interaction networks of gene families have become a very useful method to study their function (Tohge and Fernie, 2010). In order to identify potential interaction networks and biological function of cassava MAPKs, we created interaction networks of MPK3, MPK4, and MPK6 based on experimentally validated interactions in Arabidopsis using STRING software. There were 10 high confidence interactive proteins involved in MPK3, MPK4, and MPK6 networks, respectively, including MEK1, WRKY33, MKKs, and PP2Cs (Figure S5, Table S6). It has been reported that these interactions might function in the regulation of developmental processes, innate immunity, hormone signaling, and abiotic stress responses (Hadiarto et al., 2006; Schweighofer et al., 2007; Qiu et al., 2008a,b; Kosetsu et al., 2010; Kim et al., 2011; Mao et al., 2011; Xie et al., 2012).

**Figure 5:**
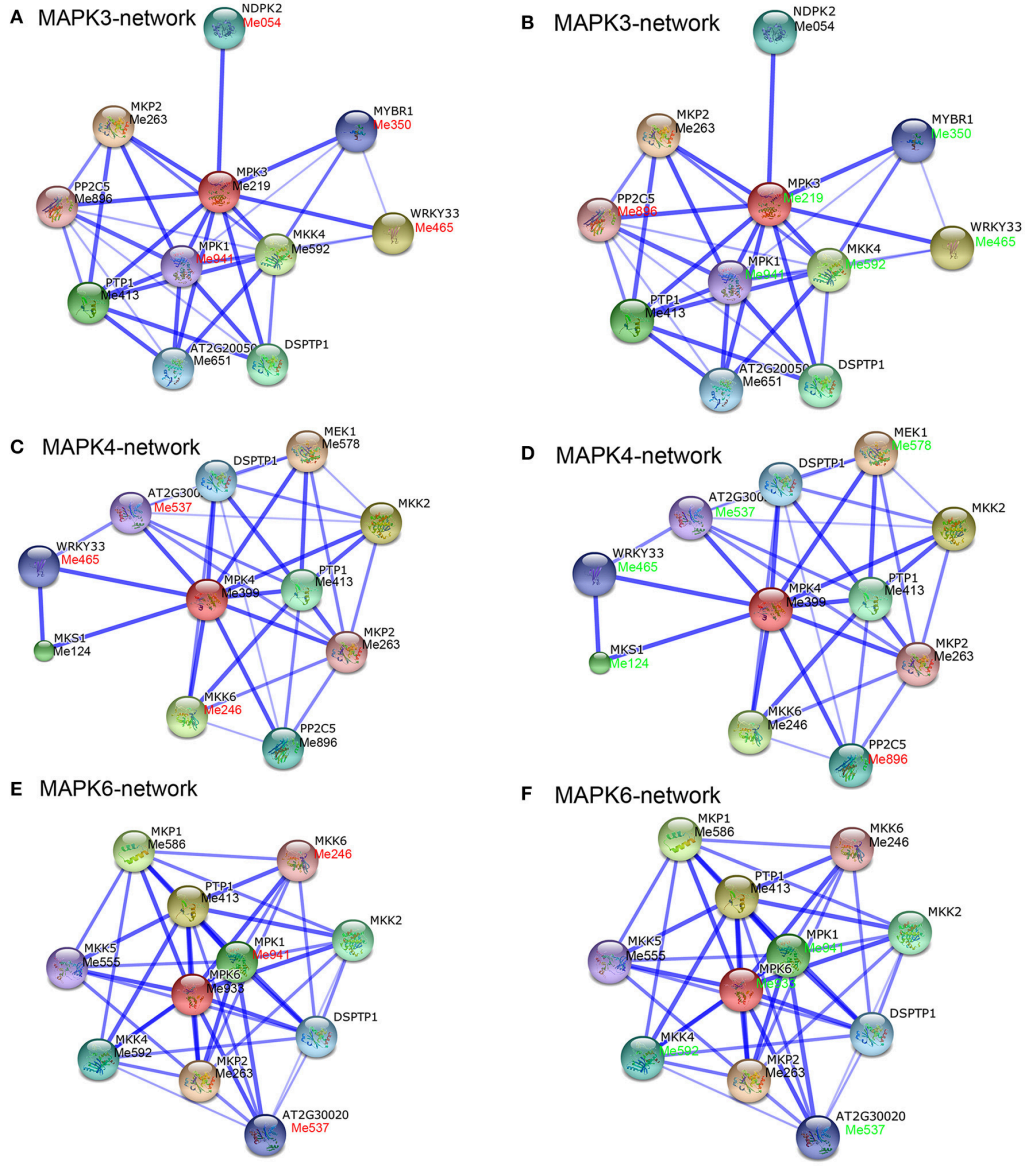
**Interaction network and co-expression analyses of ***MAPK*** genes in leaves of Arg7 (A,C,E) and W14 (B,D,F) and related genes in Arabidopsis**. The homologous genes of cassava are in parentheses. The genes marked with red font show upregulation based on 2-fold change. The genes marked with green font show downregulation based on 2-fold change.

**Figure 6:**
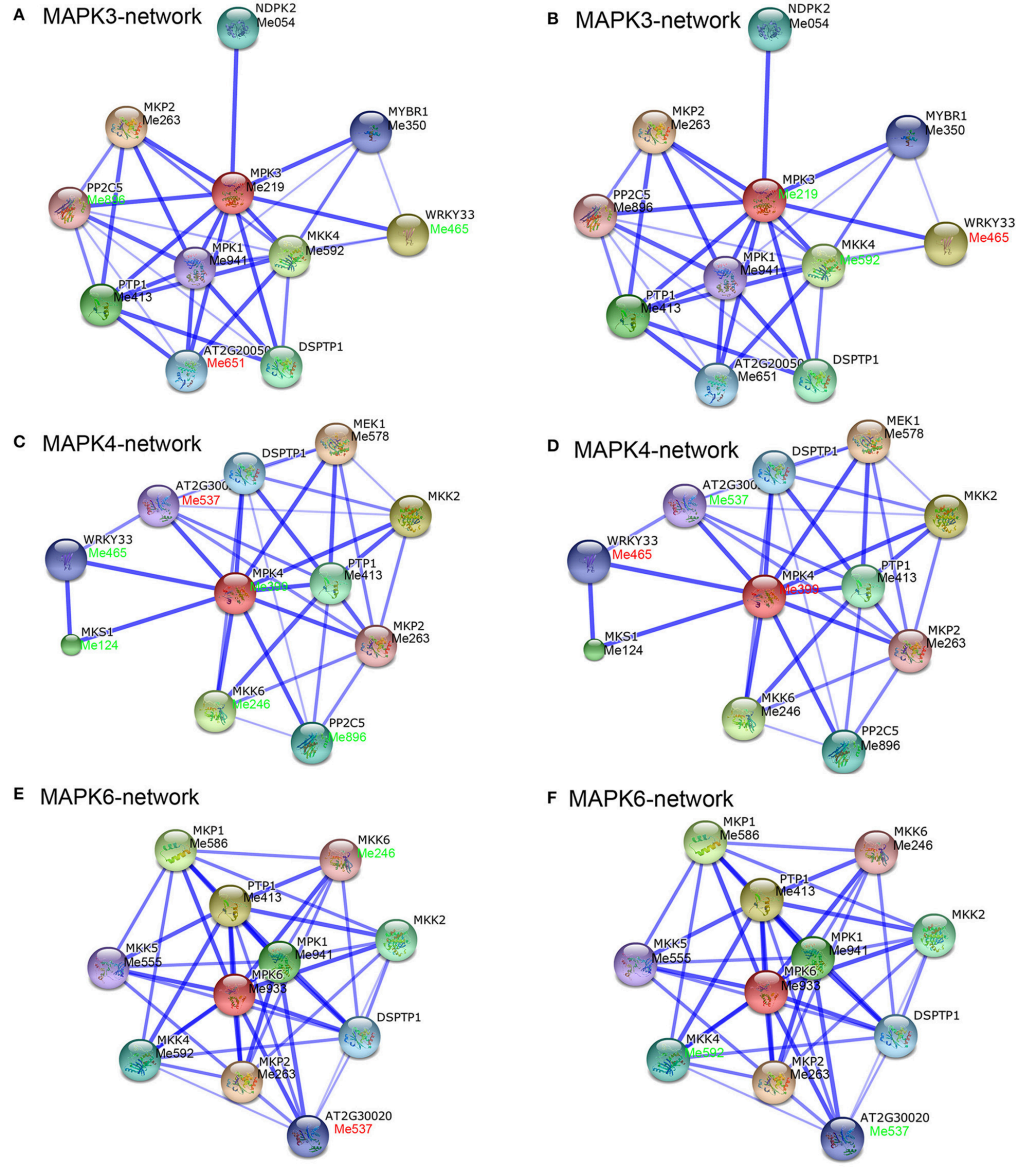
**Interaction network and co-expression analyses of ***MAPK*** genes in roots of Arg7 (A,C,E) and W14 (B,D,F) and related genes in Arabidopsis**. The homologous genes of cassava are in parentheses. The genes marked with red font show upregulation based on 2-fold change. The genes marked with green font show downregulation based on 2-fold change.

In addition, we have identified homologs of these proteins in cassava by using reciprocal Blastp analyses, and the corresponding expression patterns of these genes in leaves and roots of Arg7 and W14 under drought treatment were obtained from the transcriptomic data (Figures 5, 6, Table S7). In leaves of Arg7, there were no significant changes at transcriptional levels for MPK3:Me219, MPK4:Me399, and MPK6:Me933 genes after drought treatment (Figures 5A,C,E). In contrast, in leaves of W14, MPK3:Me219-MKK4:Me592, MPK3:Me219-MYBR1:Me350, and MPK3:Me219-WRKY33:Me465 in the MPK3 network (Figure 5B), and MPK6:Me933-MKK4:Me592 in the MPK6 network (Figure 5F) showed down-regulation. These results indicated that MPK3- and MPK6-mediated networks were inhibited by drought treatment in the leaves of W14. Previous studies demonstrated that MPK3, MPK6, and MKK4 could be activated by drought or osmotic stress (Kim et al., 2011; Tsugama et al., 2012). WRKY33 and MYBR1, as the downstream components of MPK3, were also involved in stress and ABA responses. WRKY33 functions on positively regulating osmotic and heat tolerances and ABA sensitivity (Jiang and Deyholos, 2009; Li et al., 2011); however, MYBR1 (MYB44) was reported to be a negative regulator of water stress, wounding, and ABA responses (Jaradat et al., 2013). These studies allow us to speculate that the repression of MKK4-MPK3 may result in compromised function of WRKY33 and MYBR1 under drought stress, thus dual regulation of W14 tolerance to drought stress.

In roots of Arg7, MPK4-mediated network mainly showed down-regulation, including MPK4:Me399-WRKY33:Me465, MPK4:Me399-MKK6:Me246, MPK4:Me399-MKS1:Me124, and MPK4:Me399- PP2C5:Me896 (Figure 6C). In contrast, MPK4:Me399-WRKY33:Me465 showed increased transcripts in roots of W14 (Figure 6D). Previously, MKS1 was reported to be multiply phosphorylated by MPK4 and may function as an adaptor linking MPK4 activity to the transcription factor WRKY33. WRKY33 appears to negatively regulate salicylic acid-mediated responses, while it positively regulates plants' tolerance to osmotic stress and ethylene-, jasmine acid-, and ABA-mediated responses (Qiu et al., 2008b; Jiang and Deyholos, 2009). Therefore, it is speculated that MPK4-mediated network may be involved in drought and multiple hormone responses in cassava roots and the activation of MPK4-WRKY33 may contribute to the strong tolerance of W14 to drought stress. Together, the interaction networks and expression patterns of MAPKs identified in this study will contribute to further functional analysis of MeMAPKs in drought stress response.

### Expression profiles of *MeMAPK* genes in response to various stresses and related signals

**Figure 7:**
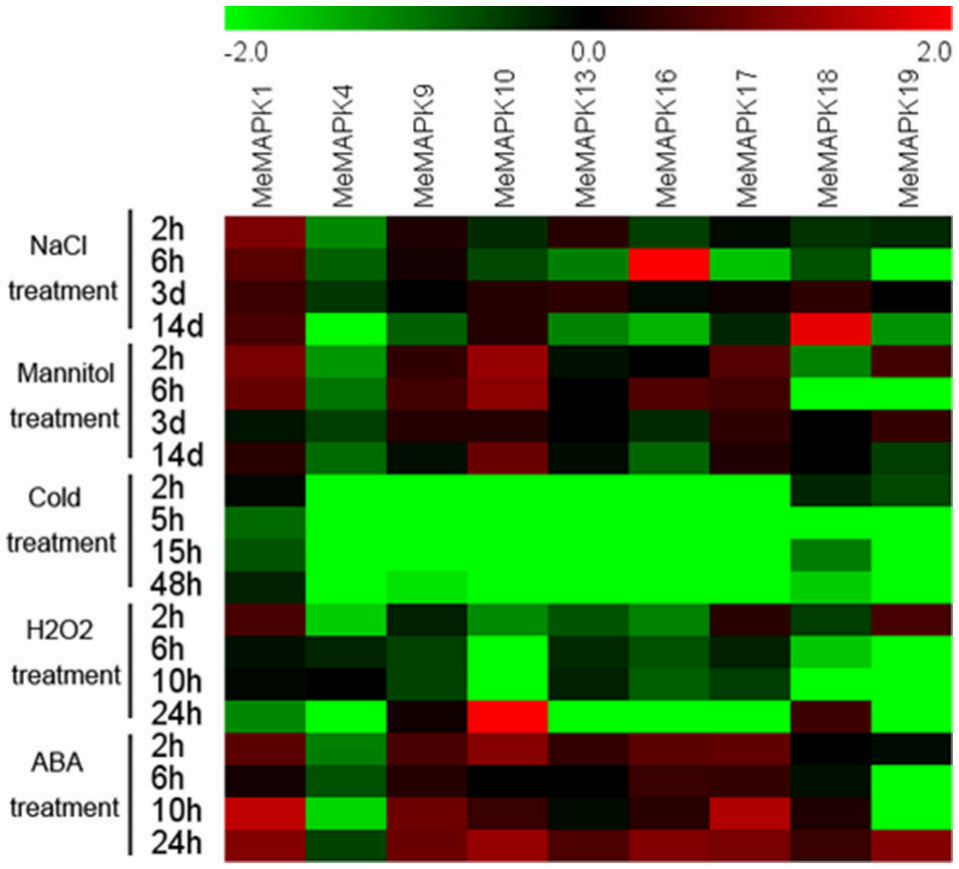
**Expression patterns of ***MAPK*** genes in leaves of cassava in response to osmotic, salt, cold, oxidative stresses, and ABA**. Log2 based value was used to create the heat map. The scale represents the relative signal intensity values.

Numerous reports have demonstrated the involvement of *MAPK* genes in abiotic stresses and related signal transduction pathways (Rodriguez et al., 2010; Chen et al., 2012; Wang et al., 2015). In the present study, based on the results of RNA-seq in different cassava accessions, 9 *MeMAPK* genes (*MeMAPK* −*1*, −*4*, −*9*, −*10*, −*13*, −*16*, −*17*, −*18*, and −*19*) distributed in the four subfamilies significantly induced by drought stress were selected for further analysis of their expression changes in response to salt, osmotic, cold, ABA, and H_2_O_2_ treatments (Figure 7, Table S8).

Salinity could causes physiological drought and ion toxicity, which interferes with plant growth. With salt treatment, *MeMAPK4* was obviously inhibited at all treated time points. *MeMAPK16, MeMAPK17*, and *MeMAPK19* were repressed at several treated time points, among which *MeMAPK16* at 14 days, *MeMAPK17* at 6 h, and *MeMAPK19* at 6 h and 14 days showed significant down-regulation. *MeMAPK10* showed significant down-regulation at 2 and 6 h, then obviously induced at 3 and 14 days. *MeMAPK9* and *MeMAPK13* were obviously inhibited at 14 days treatment. *MeMAPK18* showed down-regulation during 2–6 h treatment and up-regulation during 3–14 days of treatment. Notably, *MeMAPK1* showed up-regulation at all treated time points, indicating its possible role in salt stress response (Figure S6). In recent years, lots of *MAPK* genes have been identified to be salt stress responsive, including *MPK9, MPK10, MPK11, MPK17*, and *MPK18* in Arabidopsis, and *OsMAPK4, OsMAPK5*, and *OsMAPK44* in rice (Fu et al., 2002; Xiong and Yang, 2003; Jeong et al., 2006; Khaled et al., 2008). Furthermore, overexpressing *MAPKs* in rice (such as *OsMAPK5, OsMAPK44*), Arabidopsis (such as *GhMPK17, ZmSIMK1*) and tobacco (such as *ZmMPK5, GhMPK2*) could greatly improve salt tolerance of transgenic plants (Xiong and Yang, 2003; Jeong et al., 2006; Gu et al., 2010; Zhang et al., 2011, 2014a,b). Nonetheless, some *MAPKs* were reported as having negative regulatory roles in salt tolerance, such as the *OsMAPK33* gene (Lee et al., 2011). These results indicate that *MAPK* family genes might be positively or negatively involved in the salt stress response.

Osmotic stress could constrict development and interfere nutrient availability when the soil dries. Under osmotic treatment, *MeMAPK10* and *MeMAPK17* showed up-regulation at all the treated time points with *MeMAPK10* exhibiting significant induction at 2, 6 h, and 14 days of treatments. *MeMAPK1* and *MeMAPK9* were both obviously induced at 2 and 6 h. *MeMAPK19* showed significant induction at 2 h and 3 days of treatment, whereas obvious repression at 6 h. *MeMAPK16* was down-regulated at 3 and 14 days treatments, but significantly up-regulated at 6 h. *MeMAPK4*, and *MeMAPK18* were mostly down-regulated at several time points, among which *MeMAPK4* was obviously inhibited during all the treated time points and *MeMAPK18* was significantly inhibited at 2 and 6 h (Figure S7). Several studies have demonstrated that *MAPK* genes play a positive role in osmotic stress, including *GhMPK17, SpMPK3, GhMPK2*, and *CsNMAPK* (Xu et al., 2010; Zhang et al., 2011, 2014b; Li et al., 2014). On the other hand, some *MAPKs*, including *AtMPK4* and *GhMPK6a*, were reported to negatively regulate osmotic stress tolerance (Droillard et al., 2004; Li et al., 2013). Based on these results, we speculated that *MeMAPKs* might play an important role in osmotic stress response.

Temperature is also a fundamental abiotic stress in the process of plant growth. Low temperature can restrain many kinds of physiological activities in plants, including germination, growth, and metabolic activities (Roberts, 1988). Although cassava has high tolerance to drought and sterile soil, it is however extremely sensitive to cold. Therefore, there is a need to study *MAPK*-mediated cold response in cassava. In response to cold stress, our results indicated that all of the 9 *MeMAPK* genes were significantly down-regulated at all the treated time points except for *MeMAPK1* that was significantly down-regulated only at 5, 15, and 48 h of treatment (Figure S8). These results were similar with the previous reports in cucumber, in which the majorities of *MAPK* genes (except for *CsMPK3, CsMPK7*, and *CsMPK13*) were down-regulated under cold treatment (Wang et al., 2015). However, some *MAPKs* were reported to be induced by cold stress, such as *OsMPK3, OsMAPK4, ZmMPK4, ZmMPK5, ZmMPK6, ZmMPK7, ZmSIMK*, and *ZmMPK17* (Fu et al., 2002; Wu et al., 2011; Pan et al., 2012; Xie et al., 2012). According a genome-wide identification and analysis of the *MAPK* gene family in *Brachypodium distachyon*, more than 56 percent of *MAPK* genes were up-regulated and none of them were down-regulated under cold treatment (Chen et al., 2012). Additionally, *ZmMPK4, SlMPK3*, and *SlMPK7* were reported to be positive regulators in response to low temperature stress (Yan et al., 2012; Yu et al., 2015a,b). These results indicate that the functions of *MAPK* genes were not conserved and *MAPK*-mediated signal transduction in cold signaling pathways was complicated in different species.

Oxidative stress is a major challenge for the growth and development of plants. H_2_O_2_ is a reactive oxygen species, which is considered as a specific component of several signaling pathways (Costa et al., 2010). To investigate the response of *MAPK* genes in H_2_O_2_ signaling pathways, we examined the expression patterns of these 9 *MAPK* genes. The results showed that all of the 9 *MeMAPK* genes were down-regulated in most of the time points after H_2_O_2_ treatment. *MeMAPK4, MeMAPK13*, and *MeMAPK16* showed down-regulation during all the time points with *MeMAPK4* obvious repression at 2 and 24 h, and *MeMAPK13* and −*16* at all the time points. *MeMAPK1* and *MeMAPK17* were obviously depressed at 24 h. *MeMAPK19* was significantly induced at 2 h, but obviously inhibited at 6, 10, and 24 h. *MeMAPK9, MeMAPK10*, and *MeMAPK18* were down-regulated during 2–10 h, then significantly up-regulated at 24 h, among which *MeMAPK9* and *MeMAPK18* were significantly down-regulated at 6 and 10 h (Figure S9). However, the majority of *MAPK* genes in *Brachypodium* are induced by H_2_O_2_ treatment (Chen et al., 2012). In Arabidopsis, *MPK9* and *MPK12* are positive regulators of ROS-mediated ABA signaling in guard cells, while some *MAPK* genes, such as *MPK6*, act as negative regulators in the oxidative stress response in Arabidopsis (Jammes et al., 2011; Jin et al., 2013). Together, these results indicate that *MAPK* genes may be involved in H_2_O_2_ signaling in cassava.

As one of the most important phytohormones, ABA, is involved in plant growth, development, and adaptation to various stress conditions (Kazuo and Kazuko, 2000; Verslues et al., 2006). Treatment with ABA induced transcriptional changes of *MPK3, MPK5, MPK6, MPK7, MPK9, MPK12, MPK18*, and *MPK20* in Arabidopsis, as well as *OsMAPK5, OsMAPK2*, and *OsMAPK44* in rice (Huang et al., 2002; Lu et al., 2002; Xiong and Yang, 2003; Jeong et al., 2006; Menges et al., 2008; Xing et al., 2008; Wang et al., 2011), suggesting a potential role of *MAPK* in ABA signaling. In our work, most *MeMAPK* genes (except *MeMAPK4* and *MeMAPK19*) were up-regulated at most time points after ABA treatment. *MeMAPK1, MeMAPK9*, and *MeMAPK17* were induced at all the treated time points with significant up-regulation at 2, 10, and 24 h. *MeMAPK10* and *MeMAPK16* showed obvious up-regulation at 2 and 24 h. *MeMAPK13* and *MeMAPK18* were significantly up-regulated at 24 h. Nevertheless, *MeMAPK4* expression was significantly depressed during all the treated time points, and *MeMAPK19* was down-regulated during 2–10 h (Figure S10). The expression patterns of *MeMAPK* genes were consistent with those of *MAPK* genes in *Brachypodium* (Chen et al., 2012). The response of *MeMAPK* genes to ABA treatment suggested that the possible roles of *MeMAPK* genes in ABA signaling.

According to these results, *MeMAPK1* was induced after osmotic, salt, and ABA treatments, whereas repressed after cold treatment. *MeMAPK4* showed repression under all these treatments. *MeMAPK9*, −*10*, −*17* were up-regulated by osmotic and ABA treatments, but down-regulated by cold treatment. *MeMAPK13* and *MeMAPK16* showed induction after ABA treatment, but repression after H_2_O_2_ and cold treatments. *MeMAPK18* and *MeMAPK19* did not show obvious trend with each of the treatment. These results indicated that a single *MeMAPK* gene may participate in various stresses or signals responses, suggesting possible roles of *MeMAPKs* in multiple signaling pathways in cassava (Figure 7).

### Validation of the differentially expressed *MeMAPK* genes by qRT-PCR analysis

According to the transcriptomic data, *MeMAPK4*, −*10*, and −*16* showed low expression levels in most tissues; *MeMAPK9*, −*13*, and −*19* had high transcriptional abundance in most tissues; and *MeMAPK4*, −*9*, −*10*, −*13*, −*16*, and −*19* were upregulated by drought in several tissues. To validate the transcriptomic data, these six genes were selected for qRT-PCR analysis. After normalization, we found that the majority of tested points (58/72, 80.5%) of these genes in different tissues and in response to drought stress showed the same trend and consistent results between RNA-seq data and qRT-PCR data (Figure S11). These results indicate that RNA-seq data are suitable for supplying the expression patterns of *MeMAPK* genes.

## Conclusion

In this study, we identified 21 *MAPK* genes from the cassava genome and established their classification and phylogeny using phylogenetic, gene structure, and conserved protein motif analyses. Expression analyses indicated the functional diversity of *MeMAPKs* in tissue development and in response to drought stress between wild subspecies and cultivated varieties and the involvement of *MeMAPKs* in response to abiotic stress and related signaling. Furthermore, interaction network and co-expression analyses of MPK3, MPK4, and MPK6 in W14 and Arg7 revealed the differential response of cassava MAPK-mediated networks in wild subspecies and cultivated varieties. This study facilitates further functional characterization of *MAPK* gene family in cassava and provides a solid foundation for the investigation of the molecular mechanism underlying the high tolerance to drought of cassava, thereby advancing our understanding of the molecular basis of genetic enhancements to cassava.

## Availability of supporting data

The cassava MAPK genes identified in this study was submitted to GenBank and the accession number was listed in Table S3. The transcriptomic data was submitted to NCBI and the accession number was listed in Table S9.

## Author contributions

WH conceived the study. YY, WH, LW, ZD, WT, XD, CZ, YW, and HZ performed the experiments and carried out the analysis. WH, YY, MP, and LW designed the experiments and wrote the manuscript. All authors read and approved the final manuscript.

### Conflict of interest statement

The authors declare that the research was conducted in the absence of any commercial or financial relationships that could be construed as a potential conflict of interest.

## Supplementary material

Table S1 **Primers used in qRT-PCR analysis**.

Table S2 **Characteristics of MAPKs in cassava**.

Table S3 **The accession numbers of MAPKs in cassava, Arabidopsis, rice, tomato, apple, and cucumber**.

Table S4 **The expression profiles of the cassava ***MAPK*** genes in different tissues**.

Table S5 **The expression profiles (log2-based values) of the cassava ***MAPK*** genes after drought treatment**.

Table S6 **Annotation summary of genes involved in the MeMAPKs interaction network**.

Table S7 **Expression patterns of the genes involved in MAPK-mediate interaction networks responding to drought in cassava**.

Table S8 **The expression profiles (log2-based values) of the cassava ***MAPK*** genes after various abiotic stress and ABA treatments**.

Table S9 **The accession number of transcriptomic data in NCBI**.

Figure S1 **Multiple sequence alignment of cassava MAPKs**.

Figure S2 **The TEY/TDY motifs identified by multiple sequence alignment analysis**. The TEY/TDY motifs was boxed with blue.

Figure S3 **Conserved motifs of cassava MAPK proteins**.

Figure S4 **Expression analyses of ***MAPK*** genes in different tissues of wild subspecies (W14) and cultivated variety (Arg7)**. FPKM value was used to create the heat map with clustering. The scale represents the relative signal intensity of FPKM values.

Figure S5 **Interaction network of AtMAPKs identified by STRING**.

Figure S6 **Expression analysis of ***MeMAPK*** genes in leaves after salt treatment**. The relative expression levels of *MeMAPK* genes in each treated time point were compared with that in each time point at normal conditions. NTC (no treatment control) at each time point was normalized as “1.” Data are means ± SE calculated from three biological replicates. Values with the same letter were not significantly different according to Duncan's multiple range tests (*P* < 0.05, *n* = 3).

Figure S7 **Expression analysis of ***MeMAPK*** genes in leaves after osmotic treatment**. The relative expression levels of *MeMAPK* genes in each treated time point were compared with that in each time point at normal conditions. NTC (no treatment control) at each time point was normalized as “1.” Data are means ± SE calculated from three biological replicates. Values with the same letter were not significantly different according to Duncan's multiple range tests (*P* < 0.05, *n* = 3).

Figure S8 **Expression analysis of ***MeMAPK*** genes in leaves after cold treatment**. The relative expression levels of *MeMAPK* genes in each treated time point were compared with that in each time point at normal conditions. NTC (no treatment control) at each time point was normalized as “1.” Data are means ± SE calculated from three biological replicates. Values with the same letter were not significantly different according to Duncan's multiple range tests (*P* < 0.05, *n* = 3).

Figure S9 **Expression analysis of ***MeMAPK*** genes in leaves after oxidative treatment**. The relative expression levels of *MeMAPK* genes in each treated time point were compared with that in each time point at normal conditions. NTC (no treatment control) at each time point was normalized as “1.” Data are means ± SE calculated from three biological replicates. Values with the same letter were not significantly different according to Duncan's multiple range tests (*P* < 0.05, *n* = 3).

Figure S10 **Expression analysis of ***MeMAPK*** genes in leaves after ABA treatment**. The relative expression levels of *MeMAPK* genes in each treated time point were compared with that in each time point at normal conditions. NTC (no treatment control) at each time point was normalized as “1.” Data are means ± SE calculated from three biological replicates. Values with the same letter were not significantly different according to Duncan's multiple range tests (*P* < 0.05, *n* = 3).

Figure S11 **Relative expression levels of 6 ***MeMAPK*** genes in different tissues and in response to drought stress were examined by qRT-PCR analysis. (A)** Expression profiles of *MAPK4*, −*9*, −*10*, −*13*, −*16*, and −*19* in different tissues of Arg7 and W14. The mRNA fold difference was relative to that of Arg7 stem samples as calibrator; **(B)** expression profiles of *MAPK4*, −*9*, −*10*, −*13*, −*16*, and −*19* in response to drought stress in Arg7, SC124, and W14. The mRNA fold difference was relative to that of untreated samples as calibrator. Data are means ± SE calculated from three biological replicates. Values with the same letter were not significantly different according to Duncan's multiple range tests (*P* < 0.05, *n* = 3).
