# Supplementary material for: Genome-Wide Identification and Expression Analysis of the Mitogen-Activated Protein Kinase Gene Family in Cassava
Source: Front Plant Sci. 2016 Aug 30;7:1294. doi: 10.3389/fpls.2016.01294 (PMC5003926; doi:10.3389/fpls.2016.01294)
Supplement: Figure S1 — Multiple sequence alignment of cassava MAPKs. [file Image1.PDF]

```
MeMAPK17   ........................................   0
MeMAPK18   ........................................   0
MeMAPK2    ........................................   0
MeMAPK20   ........................................   0
MeMAPK7    ........................................   0
MeMAPK10   ........................................   0
MeMAPK16   ........................................   0
MeMAPK8    ........................................   0
MeMAPK1    ........................................   0
MeMAPK14   ........................................   0
MeMAPK4    ........................................   0
MeMAPK6    ........................................   0
MeMAPK13   ........................................   0
MeMAPK21   ........................................   0
MeMAPK9    ........................................   0
MeMAPK19   ........................................   0
MeMAPK5    MGSGTFVDGVRRWFQRRNTSASSSSASNNFVNG.ADTDLS  39
MeMAPK15   MGRGNLVDGVRRWFQRRNTSSSSVSSSNTVNCGDNNNNIS  40
MeMAPK11   ........................................   0
MeMAPK3    ........................................   0
MeMAPK12   ........................................   0
Consensus

MeMAPK17   ........................................   0
MeMAPK18   ........................................   0
MeMAPK2    ........................................   0
MeMAPK20   ........................................   0
MeMAPK7    ........................................   0
MeMAPK10   ........................................   0
MeMAPK16   ........................................   0
MeMAPK8    ........................................   0
MeMAPK1    ..................MDGGRPGQPADTDMEDAAAAAA  22
MeMAPK14   ........................................   0
MeMAPK4    ........................................   0
MeMAPK6    ........................................   0
MeMAPK13   ........................................   0
MeMAPK21   ........................................   0
MeMAPK9    ........................................   0
MeMAPK19   ........................................   0
MeMAPK5    GHPQSSATSVHERGRKEEGGE....LENQLTVIEDFDFSG  75
MeMAPK15   GQPQSSATVVRERLSEEGVGEQQLEEENQLKVIEDFDFSG  80
MeMAPK11   ........................................   0
MeMAPK3    ........................................   0
MeMAPK12   ........................................   0
Consensus
```

```
MeMAPK17   ........MSMESSSGSSEHNVRRIPTHGGRYVQYNVYGN    32
MeMAPK18   ........MSMESSSGSSEHNVRRIPTHGGRYVQYNVYGN    32
MeMAPK2    ..........MESSSGSGEHNVRGIPTHGGRYVQYNVYGN    30
MeMAPK20   ........................................     0
MeMAPK7    MATNNKDSSSASTADAAHGTKVKGVPTHGGRYVQYNVYGN    40
MeMAPK10   ....MAASSSKDSTEGAHPTKIKGVPTHGGRYVQYNVYGN    36
MeMAPK16   ............MENESMVIEDKGITSYGGRYLQYNILGS    28
MeMAPK8    ................MANGEFPAVPTHGGQFIQYDIFGN    24
MeMAPK1    PPPSDPHQQQQQVSPSGGIENIPATLSHGGRFIQYNIFGN    62
MeMAPK14   ........................................     0
MeMAPK4    ..............MATPVEPPNGVRIQGKHY..YSMWQT    24
MeMAPK6    ..............MATPVEPPNGVRIQGKHY..YSMWQT    24
MeMAPK13   ........................................     0
MeMAPK21   ......................MQQDHWKKNSVEMDFFSE    18
MeMAPK9    ...............................MLEKGFFTE     9
MeMAPK19   ...............................MLEKEFFTE     9
MeMAPK5    LKHIRVPKRSTHFAFG....STTTIGPPKKGSAEAEFFTE   111
MeMAPK15   LKHIKIPKRNTHFAFGFAATTTTTMDAHKKGSAEADFFTE   120
MeMAPK11   ......................MQPDQRKKSSVDVDFFTE    18
MeMAPK3    ......................MQPDQRKKSSVDVDFFTQ    18
MeMAPK12   ......................MQPDQRKKSSVDVDFFTQ    18
Consensus

MeMAPK17   LFEVSSKYVPPIRPVGRGAYGIVCAAMNSETQEEVAIKKI    72
MeMAPK18   LFEVSSKYVPPIRPVGRGAYGIVCAAMNSETQEEVAIKKI    72
MeMAPK2    LFEVSRKYVPPIRPVGRGAYGIVCAAINSETREEVAIKKI    70
MeMAPK20   ........................................     0
MeMAPK7    LFEVSSKYVPPIRPIGRGAYGIVCAAVNSETREEVAIKKI    80
MeMAPK10   LFEVSSKYVPPIRPIGRGSIGIVCAAMNSETREEVAIKKI    76
MeMAPK16   LFEVSSKYVPPIQPVGRGAYGIVCCARNAETKEEVAIKKI    68
MeMAPK8    LFEITSKYQPPIMPIGRGAYGIVCSVLNSETNEMVAIKKI    64
MeMAPK1    IFEVTAKYKPPIMPIGKGAYGIVCSALNSETGEHVAIKKI   102
MeMAPK14   ....................................MLLI     4
MeMAPK4    LFEIDTKYVP.IKPIGRGAYGIVCSSVNRETNEKVAIKKI    63
MeMAPK6    LFEIDTKYVP.IKPIGRGAYGIVCSSVNKETNEKVAIKKI    63
MeMAPK13   ........................................     0
MeMAPK21   YGDANRYKIQ..EVIGKGSYGVVCSAIDTYTGEKVAIKKI    56
MeMAPK9    YGEANQYEIQ..EVVGKGSYGVVASAIDTHTGEKVAIKKM    47
MeMAPK19   YGEANQYEIQ..EVIGKGSYGVVASAIDTHTGEKVAIKKM    47
MeMAPK5    YGEASRYQIQ..EVVGTGSYGVVCSAIDTHTGEKVAIKKI   149
MeMAPK15   YGEASRYQVQ..EVVGKGSYGVVASATDTHTGEKVAIKKI   158
MeMAPK11   YGEGSRYRIE..EVIGKGSYGVVCSAYDTHIGEKVAIKKI    56
MeMAPK3    YGEGSRYRIE..EVIGKGSYGVVCSAYDTHIGEKVAIKKI    56
MeMAPK12   YGEGSRYRIE..EVIGKGSYGVVCSAYDTHIGEKVAIKKI    56
Consensus
```

```
MeMAPK17   GNAFDNRIDAKRTLREIKLLRHMDHENIVALRDIIRPPQK   112
MeMAPK18   GNAFDNRIDAKRTLREIKLLRHMDHENIVALRDIIRPPQK   112
MeMAPK2    GNAFDNRIDAKRTLREIKLLRHMSHENIIALRDIIRPPQK   110
MeMAPK20   ......................MSHENIIALRDIIRPPQK    18
MeMAPK7    GNAFDNRIDAKRTLREIKLLRHMDHENVIAVRDIIRPPNK   120
MeMAPK10   GNAFDNKIDAKRTLREIKLLRHMDHENVIAIRDIIRPPKK   116
MeMAPK16   GNAFDNRIDAKRTLREIKLLCHMDHENIIKIKDIIPPPER   108
MeMAPK8    ANAFDNHMDAKRTLREIKLLRHFDHENVVAIRDVIPPPLR   104
MeMAPK1    ANAFDNKIDAKRTLREIKLLRHMDHENVVAIRDIIPPPRR   142
MeMAPK14   IKLMPRGLSVRSSCFVIWIMKTL....VVAIRDIIPPPRR    40
MeMAPK4    HNAFENHVDALRTLRELKLLRHLRHENVIQLKDVMMPIHK   103
MeMAPK6    HNAFENRVDALRTLRELKLLRHLRNENVIQLKDVMMPIHR   103
MeMAPK13   ..................................MLPSSK     6
MeMAPK21   HDIFEHISDAARILREIKLLRLLRHPDIVEIKHIMLPPSR    96
MeMAPK9    TNIFEHVSDATRILREIKLLRLLRHPDIVEIKHIMLPPSP    87
MeMAPK19   TNIFEHVSDATRILREIKLLRLLRHPDIVEIKHIMLPPSP    87
MeMAPK5    NDVFEHVSDATRILREIKLLRLLQHPDIVEIKHIMLPPSR   189
MeMAPK15   NDVFEHVSDATRILREIKLLRLLKHPDIVEIKHIMLPPSR   198
MeMAPK11   NDIFEHVSDATRILREIKLLRLLRHPDIVEIKHILLPPSR    96
MeMAPK3    NDIFEHVSDATRILREIKLLRLLRHPDIVEIKHILLPPSR    96
MeMAPK12   NDIFEHVSDATRILREIKLLRLLRHPDIVEIKHILLPPSR    96
Consensus                                      p

MeMAPK17   ENFNDVYIVYELMDTDLHQIIRSNQPLTDDHCRYFLYQLL   152
MeMAPK18   ENFNDVYIVYELMDTDLHQIIRSNQPLTDDHCRYFLYQLL   152
MeMAPK2    ENFNDVYIVYELMDTDLHQIIRSNQPLTDDHCRYFLYQLL   150
MeMAPK20   ENFNDVYIVYELMDTDLHQIIRSNQPLTDDHCRYFLYQLL    58
MeMAPK7    EAFNDVYIVYELMDTDLHHIIRSDQQLTDDHCQYFLYQLL   160
MeMAPK10   EAFNDVYIVYELMDTNLHHFIHSDQPLTCDHCQYFLYQLL   156
MeMAPK16   ETFNDVYIVYELMDTDLHQIIRSSQTLTDDHCQYFLYQLL   148
MeMAPK8    REFTDVYIATELMDTDLHQIIRSNQGLSEEHCQYFLYQIL   144
MeMAPK1    ESFNDVYIAYELMDTDLHQIIRSNQALSEEHCQYFLYQIL   182
MeMAPK14   ESFNDVYIAYELMDTDLHQIIRSNQALSEEHCQYFLYQIL    80
MeMAPK4    RNFKDVYLVYELMDTDLHQIIKSSQALSNDHCQYFLFQLL   143
MeMAPK6    RSFKDVYLVYELMDTDLHQIIKSSQTLSNDHCQYFLFQLL   143
MeMAPK13   REFKDIFVVFELMESDLHQVIKANDDLTREHHQFFLYQML    46
MeMAPK21   RDFKDIYVVFELMESDLHQVIKANDDLTREHYQFFLYQLL   136
MeMAPK9    REFKDIYVVFELMESDLHQVIKLNDDLTPEHHQFFLYQLL   127
MeMAPK19   REFKDIYVIFELMESDLHQVIKLNDDLTPEHHQFFLYQLL   127
MeMAPK5    REFRDIYVVFELMESDLHQVIKANDDLTPEHYQFFLYQLL   229
MeMAPK15   REFRDIYVVFELMESDLHQVIKANDDLTPEHYQFFLYQLL   238
MeMAPK11   REFKDIYVVFELMESDLHQVIKANDDLTPEHYQFFLYQLL   136
MeMAPK3    REFKDIYVVFELMESDLHQVIKANDDLTPEHYQFFLYQLL   136
MeMAPK12   REFKDIYVVFELMESDLHQVIKANDDLTPEHYQFFLYQLL   136
Consensus    f d     elm   lh i      l   h   fl q l
```

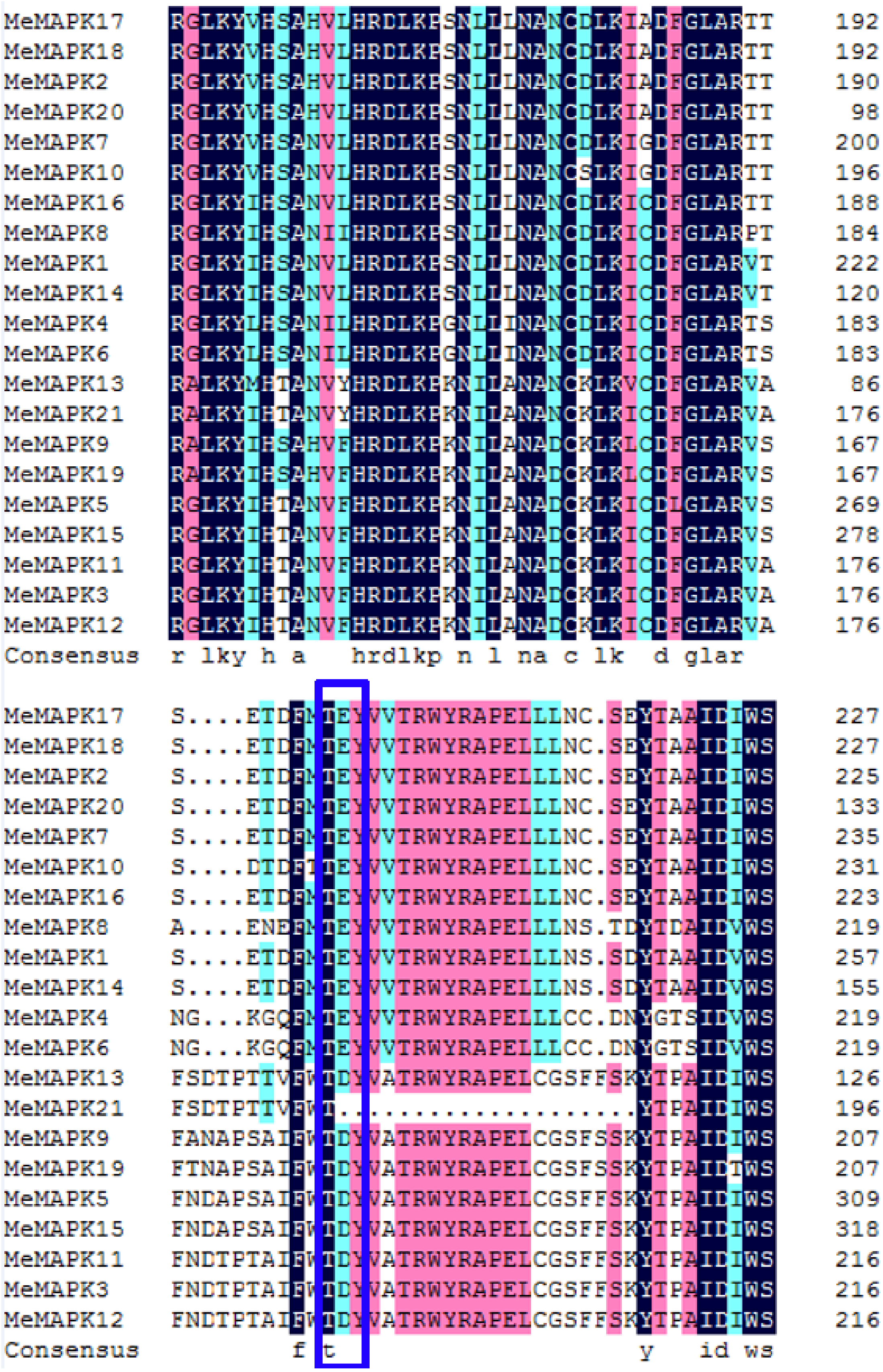
MeMAPK17 RGLKYVHSAHVLHRDLKPSNLLLNANCDLKIADFGLARTT 192
MeMAPK18 RGLKYVHSAHVLHRDLKPSNLLLNANCDLKIADFGLARTT 192
MeMAPK2 RGLKYVHSAHVLHRDLKPSNLLLNANCDLKIADFGLARTT 190
MeMAPK20 RGLKYVHSAHVLHRDLKPSNLLLNANCDLKIADFGLARTT 98
MeMAPK7 RGLKYVHSANVLHRDLKPSNLLLNANCDLKIGDFGLARTT 200
MeMAPK10 RGLKYVHSANVLHRDLKPSNLLLNANCSLKIGDFGLARTT 196
MeMAPK16 RGLKYIHSANVLHRDLKPSNLLLNANCDLKICDFGLARTT 188
MeMAPK8 RGLKYIHSANIIHRDLKPSNLLLNANCDLKICDFGLARPT 184
MeMAPK1 RGLKYIHSANVLHRDLKPSNLLLNANCDLKICDFGLARVT 222
MeMAPK14 RGLKYIHSANVLHRDLKPSNLLLNANCDLKICDFGLARVT 120
MeMAPK4 RGLKYLHSANILHRDLKPGNLLINANCDLKICDFGLARTS 183
MeMAPK6 RGLKYLHSANILHRDLKPGNLLINANCDLKICDFGLARTS 183
MeMAPK13 RALKYMHTANVYHRDLKPKNILANANCKLKVCDFGLARVA 86
MeMAPK21 RALKYIHTANVYHRDLKPKNILANANCKLKICDFGLARVA 176
MeMAPK9 RALKYIHSAHVFHRDLKPKNILANADCKLKLCDFGLARVS 167
MeMAPK19 RALKYIHSAHVFHRDLKPKNILANADCKLKLCDFGLARVS 167
MeMAPK5 RGLKYIHTANVFHRDLKPKNILANADCKLKICDLGLARVS 269
MeMAPK15 RGLKYIHTANVFHRDLKPKNILANADCKLKICDFGLARVS 278
MeMAPK11 RGLKYIHTANVFHRDLKPKNILANADCKLKICDFGLARVA 176
MeMAPK3 RGLKYIHTANVFHRDLKPKNILANADCKLKICDFGLARVA 176
MeMAPK12 RGLKYIHTANVFHRDLKPKNILANADCKLKICDFGLARVA 176
Consensus r lky h a hrdlkp n l na c lk d glar
MeMAPK17 S....ETDFMTEYVVTRWYRAPELLLNC.SEYTAAIDIWS 227
MeMAPK18 S....ETDFMTEYVVTRWYRAPELLLNC.SEYTAAIDIWS 227
MeMAPK2 S....ETDFMTEYVVTRWYRAPELLLNC.SEYTAAIDIWS 225
MeMAPK20 S....ETDFMTEYVVTRWYRAPELLLNC.SEYTAAIDIWS 133
MeMAPK7 S....ETDFMTEYVVTRWYRAPELLLNC.SEYTAAIDIWS 235
MeMAPK10 S....DTDFTTEYVVTRWYRAPELLLNC.SEYTAAIDIWS 231
MeMAPK16 S....ETDFMTEYVVTRWYRAPELLLNC.SEYTAAIDIWS 223
MeMAPK8 A....ENEFMTEYVVTRWYRAPELLLNS.TDYTDAIDVWS 219
MeMAPK1 S....ETDFMTEYVVTRWYRAPELLLNS.SDYTAAIDVWS 257
MeMAPK14 S....ETDFMTEYVVTRWYRAPELLLNS.SDYTAAIDVWS 155
MeMAPK4 NG...KGQFMTEYVVTRWYRAPELLLCC.DNYGTSIDVWS 219
MeMAPK6 NG...KGQFMTEYVVTRWYRAPELLLCC.DNYGTSIDVWS 219
MeMAPK13 FSDTPTTVFWTDYVATRWYRAPELCGSFFSKYTPAIDIWS 126
MeMAPK21 FSDTPTTVFWT...................YTPAIDIWS 196
MeMAPK9 FANAPSAIFWTDYVATRWYRAPELCGSFSSKYTPAIDIWS 207
MeMAPK19 FTNAPSAIFWTDYVATRWYRAPELCGSFSSKYTPAIDTWS 207
MeMAPK5 FNDAPSAIFWTDYVATRWYRAPELCGSFFSKYTPAIDIWS 309
MeMAPK15 FNDAPSAIFWTDYVATRWYRAPELCGSFFSKYTPAIDIWS 318
MeMAPK11 FNDTPTAIFWTDYVATRWYRAPELCGSFFSKYTPAIDIWS 216
MeMAPK3 FNDTPTAIFWTDYVATRWYRAPELCGSFFSKYTPAIDIWS 216
MeMAPK12 FNDTPTAIFWTDYVATRWYRAPELCGSFFSKYTPAIDIWS 216
Consensus f t y id ws

```
MeMAPK17    VGCILGEIMTRQPLFPGKDYVHQLRLITELIGSPDDSSLG    267
MeMAPK18    VGCILGEIMTRQPLFPGKDYVHQLRLITELIGSPDDSSLG    267
MeMAPK2     VGCILGEIMTRQPLFPGKDYVHQLRLITELIGSPDDASLG    265
MeMAPK20    VGCILGEIMTRQPLFPGKDYVHQLRLITELIGSPDDASLG    173
MeMAPK7     VGCILGEIMTREPLFPGKDYVHQLRLIIELIGSPDDASLG    275
MeMAPK10    VGCILGEIMTREPLFPGKDYVHQLRLITELIGSPDDASLG    271
MeMAPK16    VGCIFMEIIRREPLFPGKDYVQQLGLITELLGSPDDSDLG    263
MeMAPK8     VGCIFMELMNRRPLFPGNDHVHQLRLLTELLGTPTEADLG    259
MeMAPK1     VGCIFMELMDRKPLFPGRDHVHQLRLLMELIGTPSEAELE    297
MeMAPK14    VGCIFMELMDRKPLFPGRDHVHQLRLLMELIGTPSEAELE    195
MeMAPK4     VGCIFAELLGRKPIFPGTECLNQLKLIINILGSQREEDLE    259
MeMAPK6     VGCIFAELLGRKPVFPGTECLNQLKLIINILGSQREVDVE    259
MeMAPK13    IGCIFAEVLTGKPLFPGKSVVHQLDLITDLLGTPSPETIS    166
MeMAPK21    IGCIFAEVLTGKPLFPGKNVVHQLDLMTDLLGTPSLDTIS    236
MeMAPK9     IGCIFAELLAGKPLFPGKNVVHELDLITDILGTPSADSIA    247
MeMAPK19    IGCIFAELLTGKPLFPGKNVVHQLDIITDVLGTPSAESIA    247
MeMAPK5     IGCIFAEMLTGKPLFPGKNVVHQLDLMTDLLGTPPPESIA    349
MeMAPK15    IGCIFAEMLTGKPLFPGKNVVHQLDLMTDLLGTPPPESIA    358
MeMAPK11    IGCIFAELLTGKPLFPGKNVVHQLDLMTDLLGTPSPEAVA    256
MeMAPK3     IGCIFAELLTGKPLFPGKNVVHQLDLMTDLLGTPSAEAIA    256
MeMAPK12    IGCIFAELLTGKPLFPGKNVVHQLDLMTDLLGTPSAEAIA    256
Consensus    gci  e      p fpg       l        g

MeMAPK17    FLRSDNARRYVRQLPQYTRQNFAARFPNKSAGAVDLLEKM    307
MeMAPK18    FLRSDNARRYVRQLPQYTRQNFAARFPNKSAGAVDLLEKM    307
MeMAPK2     FLRSENARRYVRQLPQYPRQNFAARFPNMSAGAVNLLEEM    305
MeMAPK20    FLRSENARRYVRQLPQYPRQNFAARFPNMSAGAVNLLEEM    213
MeMAPK7     FLRSNNARRYVRQLPQYRKQNFSVRFPNMSSGAADLLEKM    315
MeMAPK10    FLRSDNARRYFRQLPKYKKQNFSVRFPNVSPGAADLLEKM    311
MeMAPK16    FLRSDNARRYVKQLPHFPKQPFAQKFPDLSAVALDLAEKM    303
MeMAPK8     FVRSEDARRYIRQLSQYPRQPLAQVFPRVNPLAIDLIDRM    299
MeMAPK1     FLN.ENAKRYIRQLPPQHRQSFTEKFPSVHPAAIDLVEKM    336
MeMAPK14    FLN.ENAKRYIRQLPPQHRQSFTEKFPSVHPAAIDLVEKM    234
MeMAPK4     FIDNPKAKKYIKSLPYSPGTPFSRLYPNAHPLAIDLLGKM    299
MeMAPK6     FIDNPKAKKYIKSLPYSPGTPFSRLYPNAHPSAIDLLQKM    299
MeMAPK13    GVRNEKARKYLTEMRKKKPVPCTLKFPNADPLALKLLQRL    206
MeMAPK21    RVRNDKARKYLTTMRKKQLVPFAQKFPNGDPLALRLLERL    276
MeMAPK9     RIGNEKARKYLNSMRKKQPIPLSKKIPNADPLALRLLERL    287
MeMAPK19    RIGNEKARKYLNSMRKKRPIPLSKKIPNADPMALRLLERL    287
MeMAPK5     RIRNEKARRYLNNMRKKLPVPFSQKFPNVDPLALRLLERL    389
MeMAPK15    RIRNEKARRYLSNMRKKQPVPFTQKFPNVDPLALSLLEKL    398
MeMAPK11    RVRNEKARRYLSSMRKKKPIPFSQKFPNADPLALRLLERM    296
MeMAPK3     RVRNEKARRYLSSMRKKKSIPFSHKFPNADPLALRLLEKM    296
MeMAPK12    RVRNEKARRYLSSMRKKKSIPFSHKFPNADPLALRLLEKM    296
Consensus          a  y                 p     a  l
```

```
MeMAPK17   IVFDPNRRITVDGALCHPYLAPLHDINEEPV...CPKPFN   344
MeMAPK18   IVFDPNRRITGNMT..........................   321
MeMAPK2    IVFDPNRRITVDAALCHPYLAPLHDINEEPV...CPRPFN   342
MeMAPK20   IVFDPNRRITVDAALCHPYLAPLHDINEEPV...CPRPFN   250
MeMAPK7    IVFDPNKRITVDEALCHPYLSSLHDINDEPV...CPRPFH   352
MeMAPK10   IVFDPNKRITVDEALCHPYLSSLHDINNEPV...CPRPFH   348
MeMAPK16   IVFDPCKRITVEEALNHPYLSSLHEINEEPT...CPSPFI   340
MeMAPK8    ITFDPTRRISVEEALAHPYLARLHDVADEPV...CPVPFT   336
MeMAPK1    ITFDPRLRITVEDALAHPYLTSLHDISDEPV...CMTPFS   373
MeMAPK14   ITFDPRLRITVEDALAHPYLTSLHDISDEPV...CMTPFS   271
MeMAPK4    IVFDPSKRITVTEALQHPYMSPLYDPSSNPP...AQVPID   336
MeMAPK6    IVFDPSKRITVTEALQHPYMSPLYDPNSNPP...AQVPID   336
MeMAPK13   LAFDPKDRPTAEEALADPYFKGLAKIEREPS.CQPISKLE   245
MeMAPK21   LAFDPKDRPTAKEALADPYFKGLAKVEREPS.CQPITKME   315
MeMAPK9    LAFDPKDRPSAEEALADPYFHDLANKEPEPS.RQPISKLE   326
MeMAPK19   LAFDPKDRPSAEEALADPYFHDLANKEHEPS.RQPISKLE   326
MeMAPK5    LAFDPKDRPTAEEALADPYFHGLSNVDREPS.TQPISKLE   428
MeMAPK15   LAFDPKDRPTAEAALAYPYFQGLANVDREPSSTQPISKLE   438
MeMAPK11   LAFEPKDRPTAEEALADPYFKGLAKVEREPS.AQPVTKME   335
MeMAPK3    LAFEPKDRPTAEEALANPYFKGLAKVEREPS.AQPVTKME   335
MeMAPK12   LAFEPKDRPTAEEALANPYFKGLAKVEREPS.AQPVTKME   335
Consensus  l f p   r

MeMAPK17   FDFEQPTFTEENIKELIWRESVKFNPDF............   372
MeMAPK18   ........................................   321
MeMAPK2    FDFEQPTFTEENIKELIWRESAKFNPDP............   370
MeMAPK20   FDFEQPTFTEENIKELIWRESAKFNPDP............   278
MeMAPK7    FDFEHPSCTEEHIKELIWRESVKFNPDPPAHWEKMNT...   389
MeMAPK10   FDFDHPSCTEEHIKELIWRESVKFNPDPPAY.........   379
MeMAPK16   FDFEQISLNEDDIKELIFAESLNFNPDVMLE.........   371
MeMAPK8    FDFEQQPLGEEQMKEMIYREALALNPEYA...........   365
MeMAPK1    FDFEQHALTEEQMKELIYREALAFNPEYQQQ.........   404
MeMAPK14   FDFEQHALTEEQMKELIYREALAFNPEYQQQ.........   302
MeMAPK4    LDIDED.LGEDMIREMMLKEILHYHPEEGTANGQMCA...   372
MeMAPK6    LDIDED.LGEEMIREMMWNEILHYHPESGAANGQMCA...   372
MeMAPK13   FEFERRRVTKEDIRELLYREILEYHPQLLKDYMNGNEGTN   285
MeMAPK21   FEFERRRVTKEDLRELIFREILEYHPQLLKDYINGTERTN   355
MeMAPK9    FEFEKRKLSEDDVRELIYREILEYHPDMLKEYLRGTDQTH   366
MeMAPK19   FEFERRKLTEDDVRELIYREILEYHPEMLKEYLRGTDHTH   366
MeMAPK5    FEFERRKLAKDDVRELIYREILEYHPQMLEEYLQGGEQTS   468
MeMAPK15   FEFERRKLAKDDI........LEYHPQMLQEYLQGGEQTS   470
MeMAPK11   FEFERRRITKEDVRELIYREILEYHPKMLKEYLEGSEPTG   375
MeMAPK3    FEFERRRITKEDVRELIYREILEYHPKMLKEYLEGSEPTG   375
MeMAPK12   FEFERRRITKEDVRELIYREILEYHPKMLKEYLEGSEPTG   375
Consensus
```

```
MeMAPK17    ........................................    372
MeMAPK18    ........................................    321
MeMAPK2     ........................................    370
MeMAPK20    ........................................    278
MeMAPK7     ........................................    389
MeMAPK10    ........................................    379
MeMAPK16    ........................................    371
MeMAPK8     ........................................    365
MeMAPK1     ........................................    404
MeMAPK14    ........................................    302
MeMAPK4     ........................................    372
MeMAPK6     ........................................    372
MeMAPK13    FLYPSAIGQFRKQFAYLEENSGR.........SAPVIPLE    316
MeMAPK21    FLYPSAVDQFRRQFAHLEENGG..........SGPVIPLE    385
MeMAPK9     FVYPSGIDRFKEQFAHLEEGNG.........RSDRISPLH    397
MeMAPK19    FVYPSGIDRFKEQFAHLEEGNG.........RSERSSPLH    397
MeMAPK5     FMYPSGVDRFKRQFAHLEEHYG.........KGERSTPLQ    499
MeMAPK15    FMYPSGVDRFKRQFAHLEENYGQGGKGGKGGKGERGTPLL    510
MeMAPK11    FMYPSAVDHFKKQFAYLEEHYG.........NGATVAPPE    406
MeMAPK3     FMYPSAVDHFKKQFAYLEEHYG.........NGATAAPPE    406
MeMAPK12    FMYPSAVDHFKKQFAYLEEHYG.........NGATAAPPE    406
Consensus

MeMAPK17    ........................................    372
MeMAPK18    ........................................    321
MeMAPK2     ........................................    370
MeMAPK20    ........................................    278
MeMAPK7     ........................................    389
MeMAPK10    ........................................    379
MeMAPK16    ........................................    371
MeMAPK8     ........................................    365
MeMAPK1     ........................................    404
MeMAPK14    ........................................    302
MeMAPK4     ........................................    372
MeMAPK6     ........................................    372
MeMAPK13    RKHVS.LPR.STVHTNTIPPNMHPSSTAFDHRHVAEDACK    354
MeMAPK21    RKHAS.LPRSTVIHANTIPPREQNIPSSRDRQTTEETYSK    424
MeMAPK9     RKHATSLPRERICTID.ETDIVIKRSSASVTRATQQSPEK    436
MeMAPK19    RKHATSLPRERICTIDDETDIVIKRSAASFTRATIQSPPK    437
MeMAPK5     RQHAS.LPRERVPAPK...EAASEKNNDLERETSTSGATN    535
MeMAPK15    RQHAS.LPRERVPAPK...EKTSEKNNDSSSQTSSSVATN    546
MeMAPK11    RQHAS.LPRPCVLYSDNTVQNSVEVTNDLSKCSIKDIEKP    445
MeMAPK3     RQHAS.LPRPCVLYSNNTVQNSAEVTNDLSKCSIKDTEKP    445
MeMAPK12    RQHAS.LPRPCVLYSNNTVQNSAEVTNDLSKCSIKDTEKP    445
Consensus
```

```
MeMAPK17    ........................................    372
MeMAPK18    ........................................    321
MeMAPK2     ........................................    370
MeMAPK20    ........................................    278
MeMAPK7     ........................................    389
MeMAPK10    ........................................    379
MeMAPK16    ........................................    371
MeMAPK8     ........................................    365
MeMAPK1     ........................................    404
MeMAPK14    ........................................    302
MeMAPK4     ........................................    372
MeMAPK6     ........................................    372
MeMAPK13    NYRAADAISGNAMKVSRPPPRVPTGCTAKPGRVVGSVVPY    394
MeMAPK21    NYRDSEGTR.INISTLQAPQRIP...LAKPGRVVGPVVPY    460
MeMAPK9     SE......STEELESANRNAVAMQTSSTKPKCSSRSLLRS    470
MeMAPK19    SETTEDLQSTEDLQSANRNAVAMQTSSTKPKCGARSLLRS    477
MeMAPK5     GP..............GNATATENGSAKGNHSTRSLLKS    560
MeMAPK15    GS..............ENAVVTQNGSSTPNRTNRSLLKS    571
MeMAPK11    HVDRSG..GIPMTRLPLQVPESIQG.AARPGKVIGSVMRY    482
MeMAPK3     RMDHSG..GIPMTRLPLQVPQIIQAGAARPGKVVGSAMRY    483
MeMAPK12    RMDHSG..GIPMTRLPLQVPQIIQG.AARPGKVVGSAMRY    482
Consensus

MeMAPK17    ........................................    372
MeMAPK18    ........................................    321
MeMAPK2     ........................................    370
MeMAPK20    ........................................    278
MeMAPK7     ........................................    389
MeMAPK10    ........................................    379
MeMAPK16    ........................................    371
MeMAPK8     ........................................    365
MeMAPK1     ........................................    404
MeMAPK14    ........................................    302
MeMAPK4     ........................................    372
MeMAPK6     ........................................    372
MeMAPK13    ENGRNIKDAYDARIFYRNAVLPPQQTVSPHCFFMNNTLTR    434
MeMAPK21    DNGSMMKDPYDPRTFVRGSFLPSQAIPGAYGYCKSSTGKQ    500
MeMAPK9     DS...ICASRCIGIVGSDREVHAL................    491
MeMAPK19    DS...ICASRCIGMVGNDREVHAL................    498
MeMAPK5     AS...ISASKCIGAQPGTDTEDTVGEVSDESVNVDLSQKV    597
MeMAPK15    AS...ISASKCIGVQSQMDIQVTN..ITLHAVII......    600
MeMAPK11    NN...CGVAAKAEALEQRRMVRNPAISTQYAAANCSYPRR    519
MeMAPK3     NN...CGVAVTAEALDQRRIVRNPAISNQYTASNCSYPRR    520
MeMAPK12    NN...CGVAVTAEALDQRRIVRNPAISNQYTASNCSYPRR    519
Consensus
```

```
MeMAPK17    ........................................    372
MeMAPK18    ........................................    321
MeMAPK2     ........................................    370
MeMAPK20    ........................................    278
MeMAPK7     ........................................    389
MeMAPK10    ........................................    379
MeMAPK16    ........................................    371
MeMAPK8     ........................................    365
MeMAPK1     ........................................    404
MeMAPK14    ........................................    302
MeMAPK4     ........................................    372
MeMAPK6     ........................................    372
MeMAPK13    GK.STESEKDTS.QAKQPECKMAAKPAPVMAIEMNANPYY    472
MeMAPK21    DRSASETQRELCSQKQVQQCGIATKYAPDIAINIDSNPFF    540
MeMAPK9     ........................................    491
MeMAPK19    ........................................    498
MeMAPK5     ATLHA...................................    602
MeMAPK15    ........................................    600
MeMAPK11    NPVCKNERGEDDGVEGSTGLQPKPQYTARKVAAAQGGTGN    559
MeMAPK3     NPVCKSERGEDEG.EGSNGLQPKPQYMARKVAAAQGGPGN    559
MeMAPK12    NPVCKSERGEDEG.EGSNGLQPKPQYMARKVAAAQGGPGN    558
Consensus

MeMAPK17    ........................................    372
MeMAPK18    ........................................    321
MeMAPK2     ........................................    370
MeMAPK20    ........................................    278
MeMAPK7     ........................................    389
MeMAPK10    ........................................    379
MeMAPK16    ........................................    371
MeMAPK8     ........................................    365
MeMAPK1     ........................................    404
MeMAPK14    ........................................    302
MeMAPK4     ........................................    372
MeMAPK6     ........................................    372
MeMAPK13    QPKA...KVEQLNERIAIDAKLLQAQSQ.......FGAAA    502
MeMAPK21    LTRAGINKVEQVDDRISINTNLLHVKAQAHAQHAGISTPA    580
MeMAPK9     ........................................    491
MeMAPK19    ........................................    498
MeMAPK5     ........................................    602
MeMAPK15    ........................................    600
MeMAPK11    HWY.....................................    562
MeMAPK3     HWY.....................................    562
MeMAPK12    HWY.....................................    561
Consensus
```

MeMAPK17	....	372
MeMAPK18	....	321
MeMAPK2	....	370
MeMAPK20	....	278
MeMAPK7	....	389
MeMAPK10	....	379
MeMAPK16	....	371
MeMAPK8	....	365
MeMAPK1	....	404
MeMAPK14	....	302
MeMAPK4	....	372
MeMAPK6	....	372
MeMAPK13	VAVAAHRNVGTVQYGLS..	519
MeMAPK21	TVAASHRKVGTVQYGMTKM	599
MeMAPK9	....	491
MeMAPK19	....	498
MeMAPK5	....	602
MeMAPK15	....	600
MeMAPK11	....	562
MeMAPK3	....	562
MeMAPK12	....	561
Consensus		
