# Supplementary figures and images for: Genome-Wide Identification and Expression Analysis of the Mitogen-Activated Protein Kinase Gene Family in Cassava
Source: Front Plant Sci. 2016 Aug 30;7:1294. doi: 10.3389/fpls.2016.01294 (PMC5003926; doi:10.3389/fpls.2016.01294)

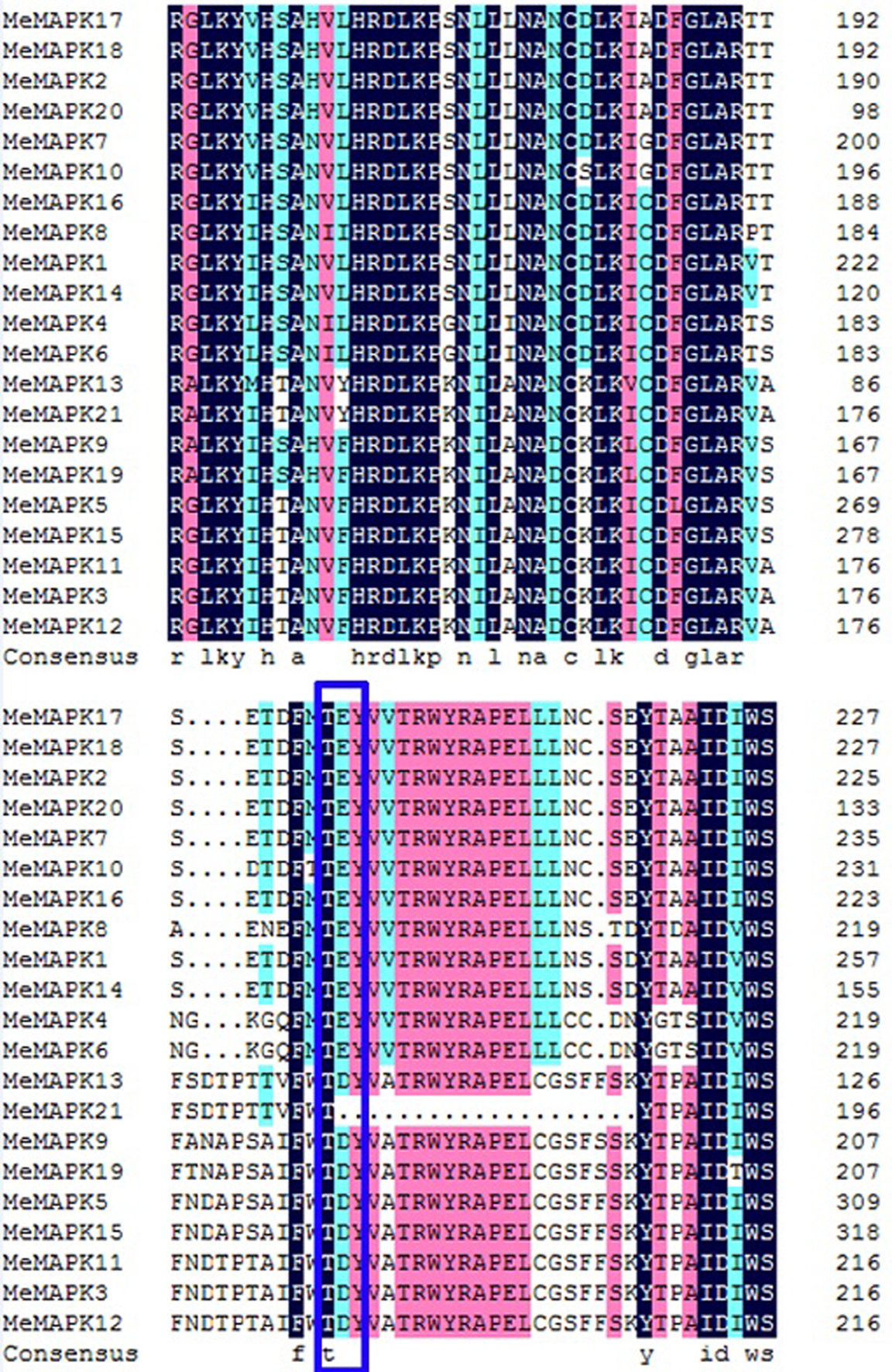

Supplement: Figure S2 — The TEY/TDY motifs identified by multiple sequence alignment analysis. The TEY/TDY motifs was boxed with blue. [file Image2.TIF]

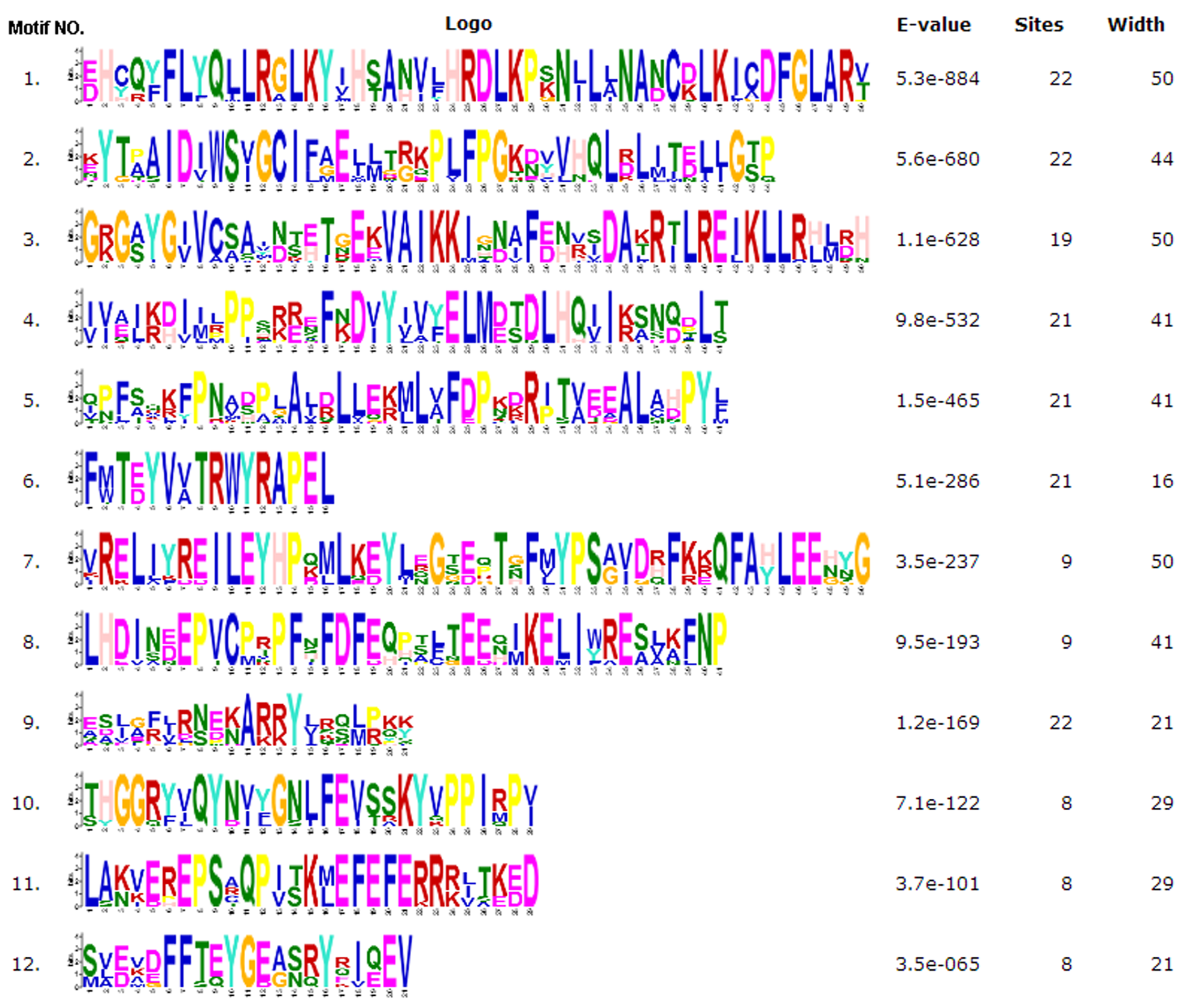

Supplement: Figure S3 — Conserved motifs of cassava MAPK proteins. [file Image3.TIF]

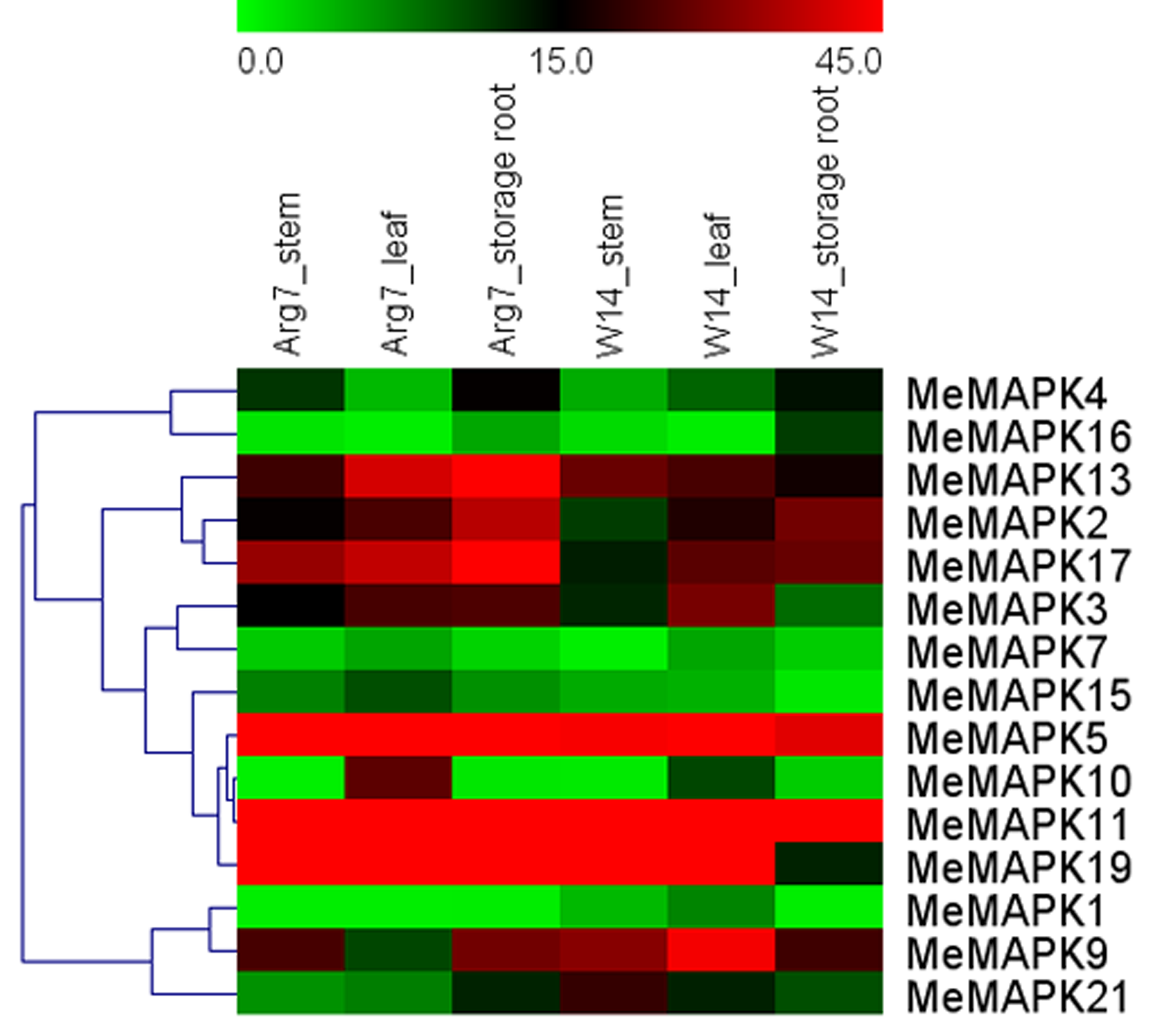

Supplement: Figure S4 — Expression analyses of MAPK genes in different tissues of wild subspecies (W14) and cultivated variety (Arg7). FPKM value was used to create the heat map with clustering. The scale represents the relative signal intensity of FPKM values. [file Image4.TIF]

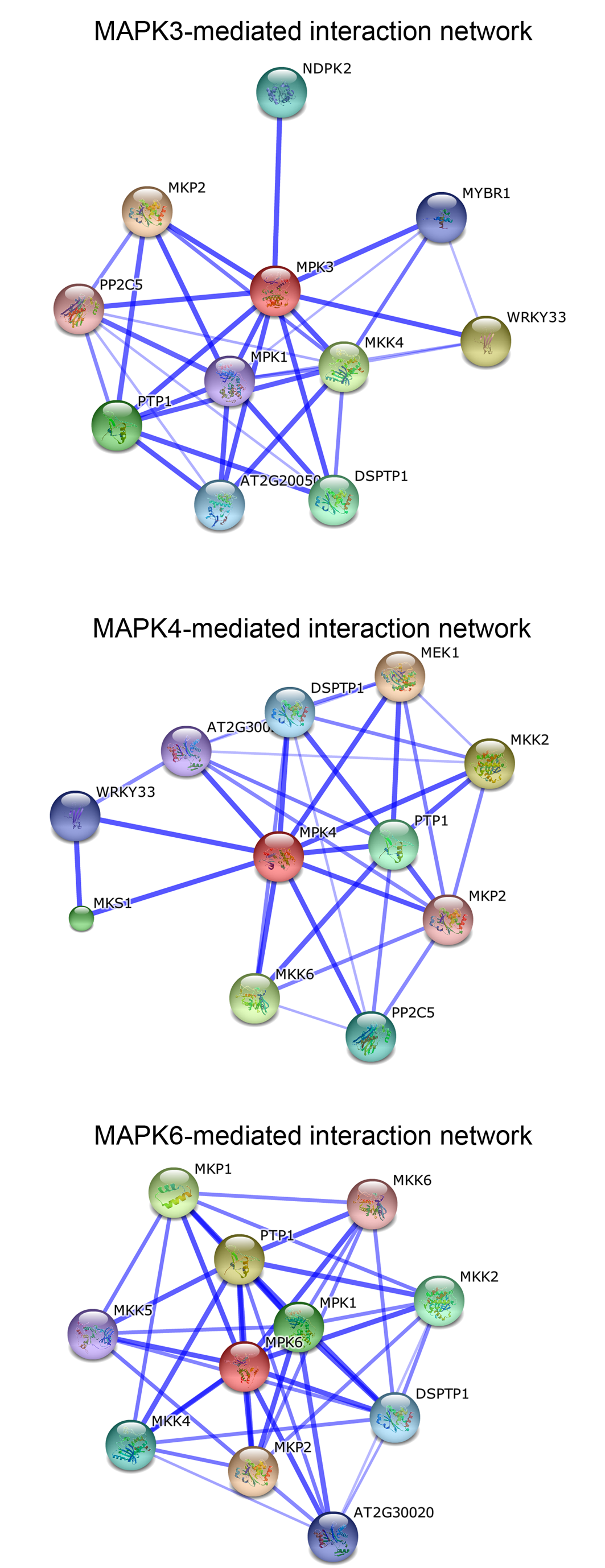

Supplement: Figure S5 — Interaction network of AtMAPKs identified by STRING. [file Image5.TIF]

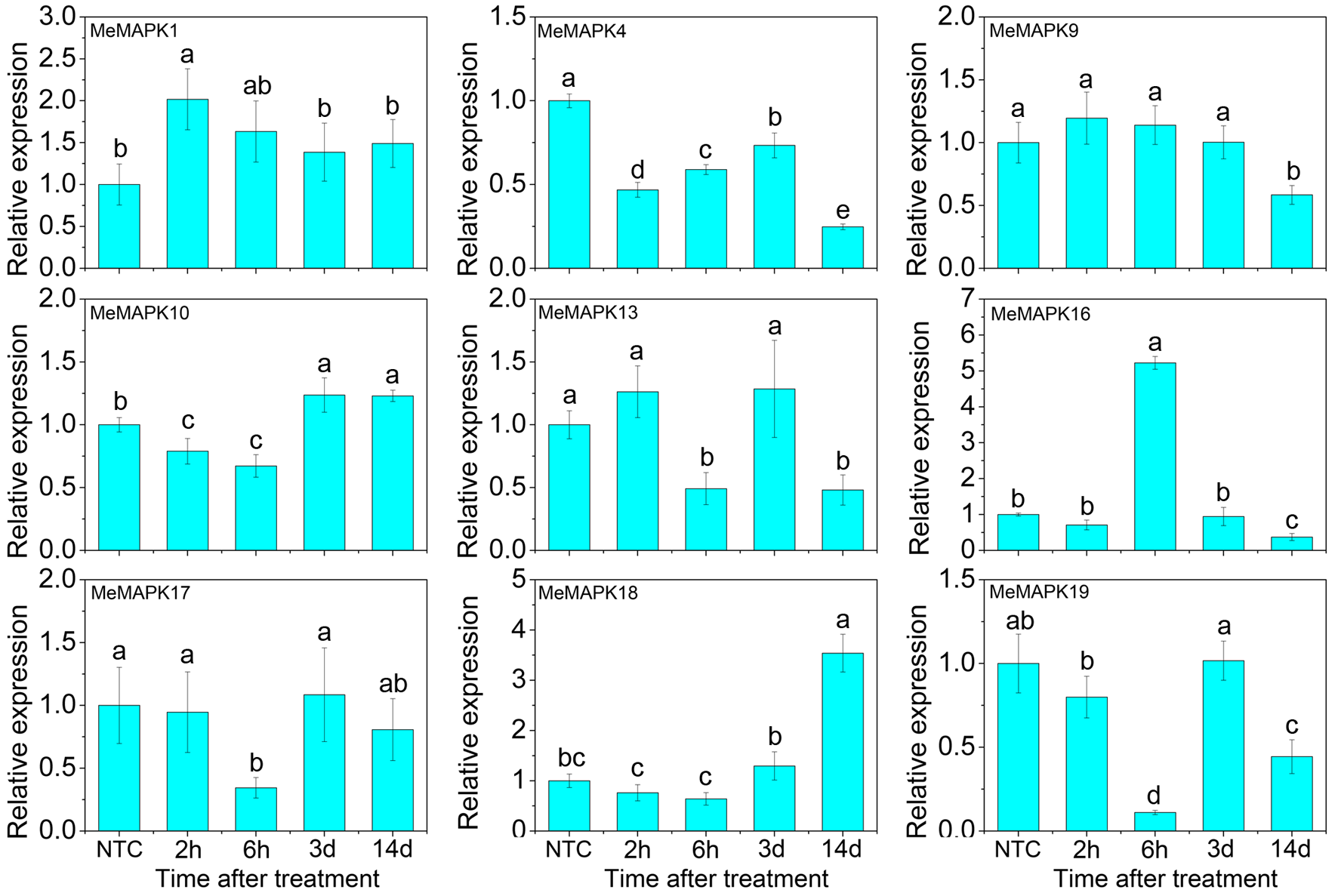

Supplement: Figure S6 — Expression analysis of MeMAPK genes in leaves after salt treatment. The relative expression levels of MeMAPK genes in each treated time point were compared with that in each time point at normal conditions. NTC (no treatment control) at each time point was normalized as “1.” Data are means ± SE calculated from three biological replicates. Values with the same letter were not significantly different according to Duncan's multiple range tests (P < 0.05, n = 3). [file Image6.TIF]

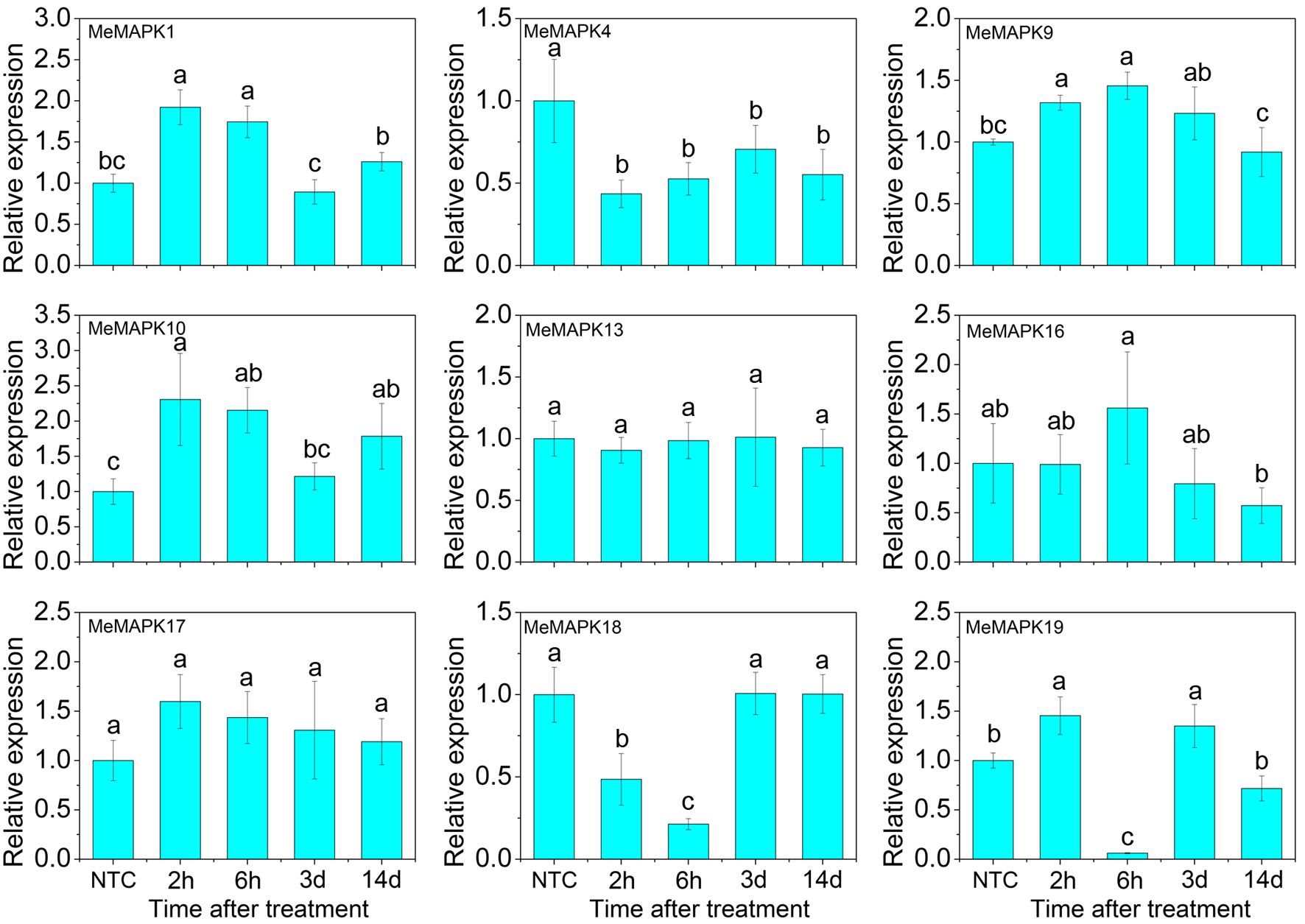

Supplement: Figure S7 — Expression analysis of MeMAPK genes in leaves after osmotic treatment. The relative expression levels of MeMAPK genes in each treated time point were compared with that in each time point at normal conditions. NTC (no treatment control) at each time point was normalized as “1.” Data are means ± SE calculated from three biological replicates. Values with the same letter were not significantly different according to Duncan's multiple range tests (P < 0.05, n = 3). [file Image7.TIF]

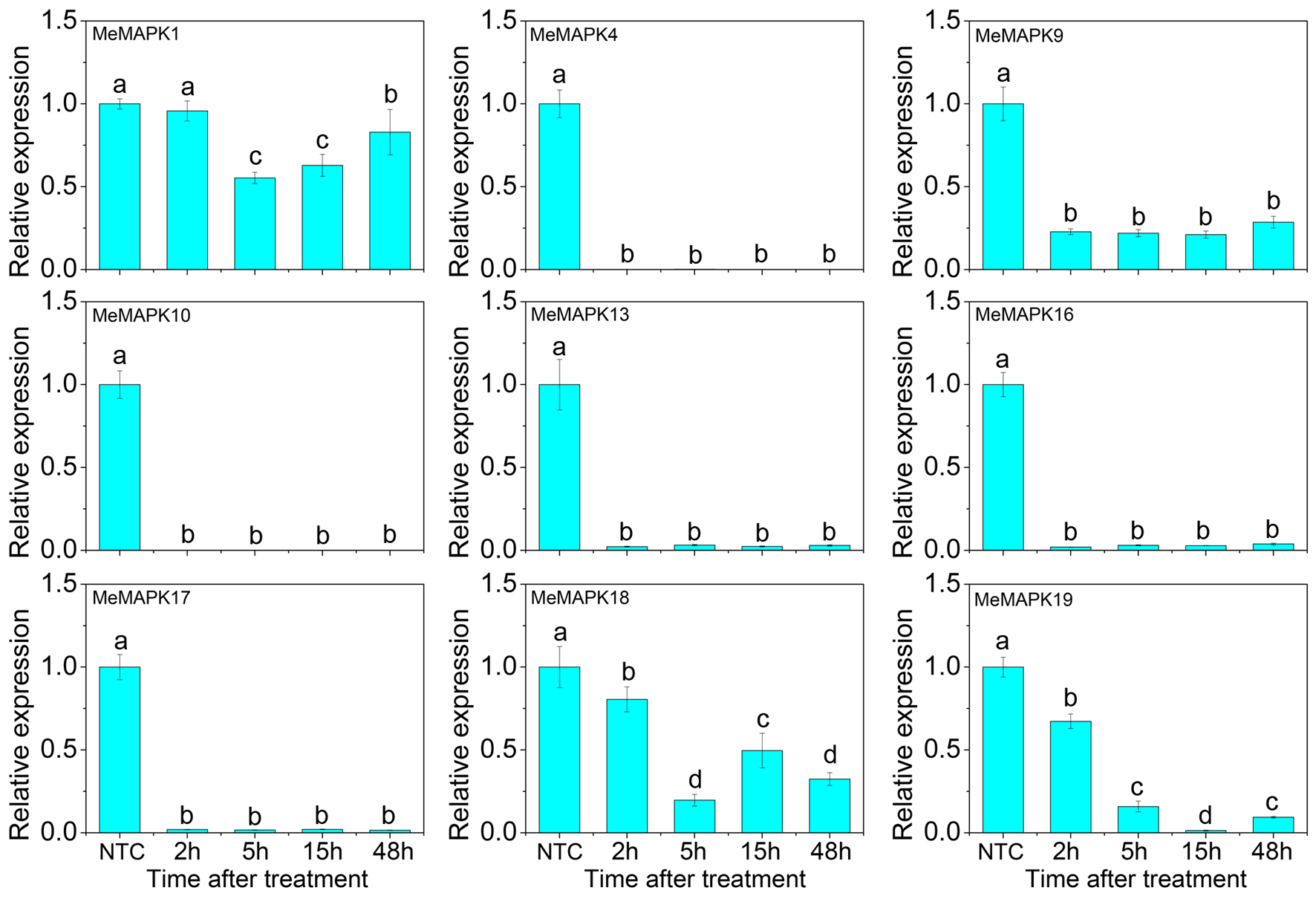

Supplement: Figure S8 — Expression analysis of MeMAPK genes in leaves after cold treatment. The relative expression levels of MeMAPK genes in each treated time point were compared with that in each time point at normal conditions. NTC (no treatment control) at each time point was normalized as “1.” Data are means ± SE calculated from three biological replicates. Values with the same letter were not significantly different according to Duncan's multiple range tests (P < 0.05, n = 3). [file Image8.TIF]

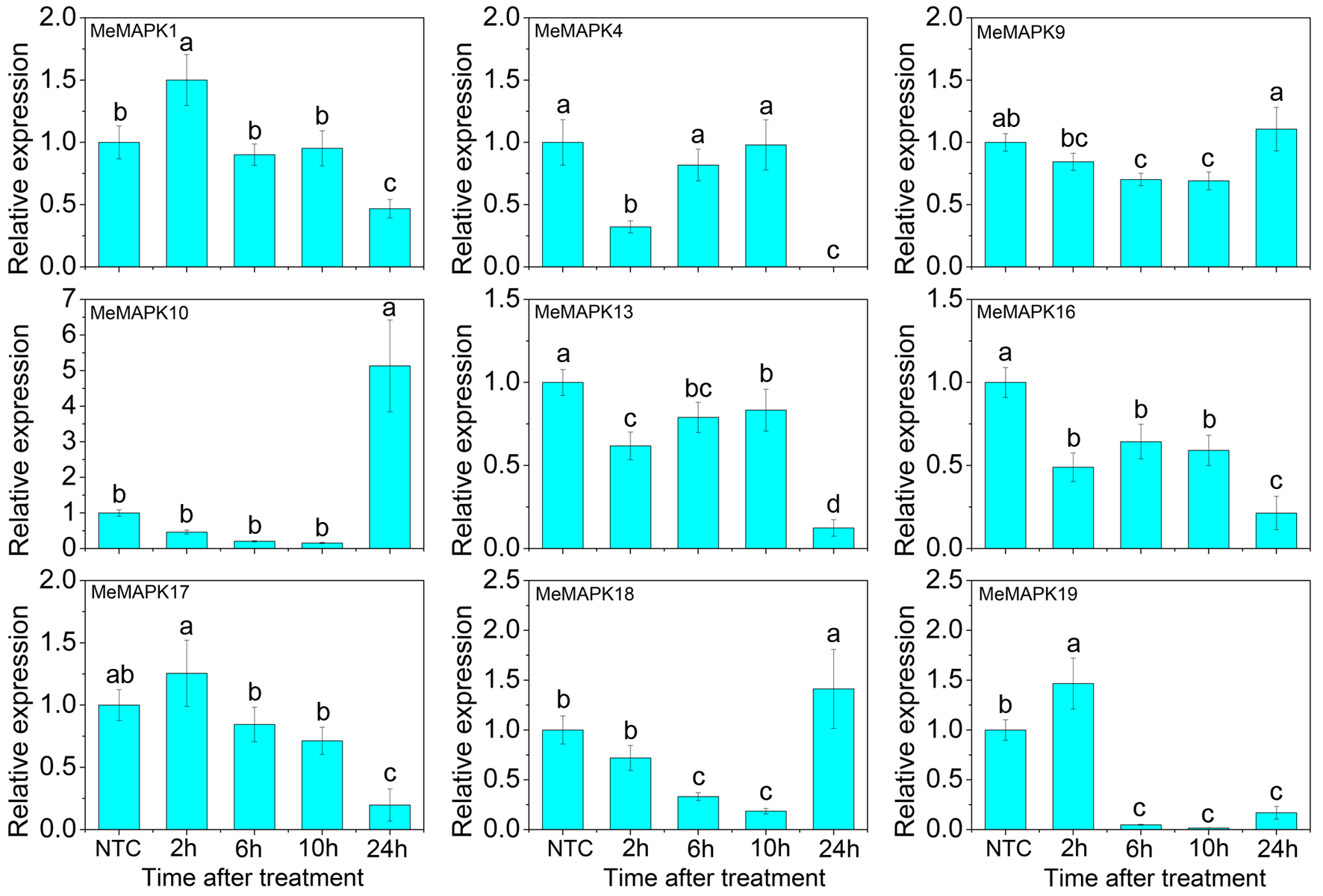

Supplement: Figure S9 — Expression analysis of MeMAPK genes in leaves after oxidative treatment. The relative expression levels of MeMAPK genes in each treated time point were compared with that in each time point at normal conditions. NTC (no treatment control) at each time point was normalized as “1.” Data are means ± SE calculated from three biological replicates. Values with the same letter were not significantly different according to Duncan's multiple range tests (P < 0.05, n = 3). [file Image9.TIF]

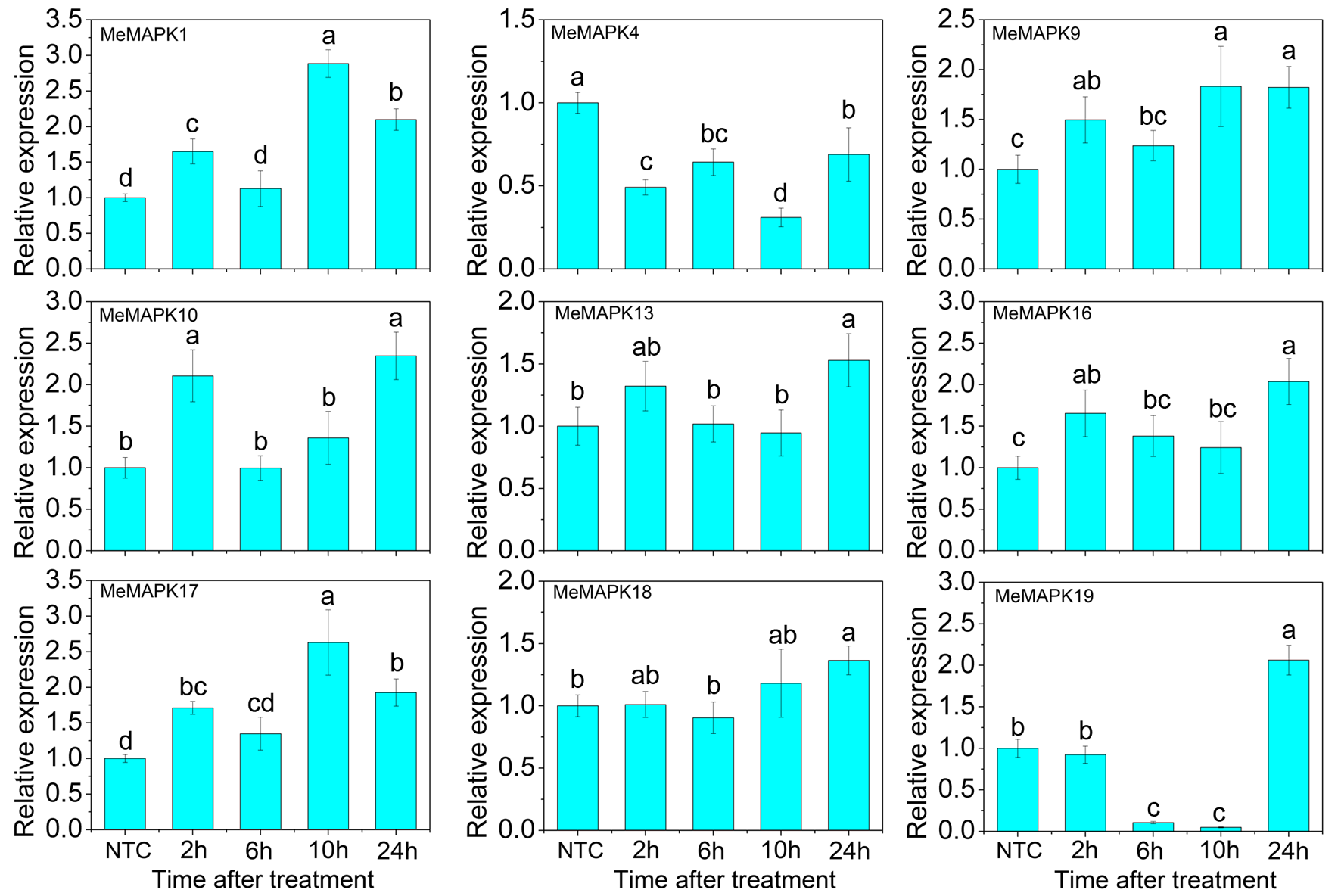

Supplement: Figure S10 — Expression analysis of MeMAPK genes in leaves after ABA treatment. The relative expression levels of MeMAPK genes in each treated time point were compared with that in each time point at normal conditions. NTC (no treatment control) at each time point was normalized as “1.” Data are means ± SE calculated from three biological replicates. Values with the same letter were not significantly different according to Duncan's multiple range tests (P < 0.05, n = 3). [file Image10.TIF]

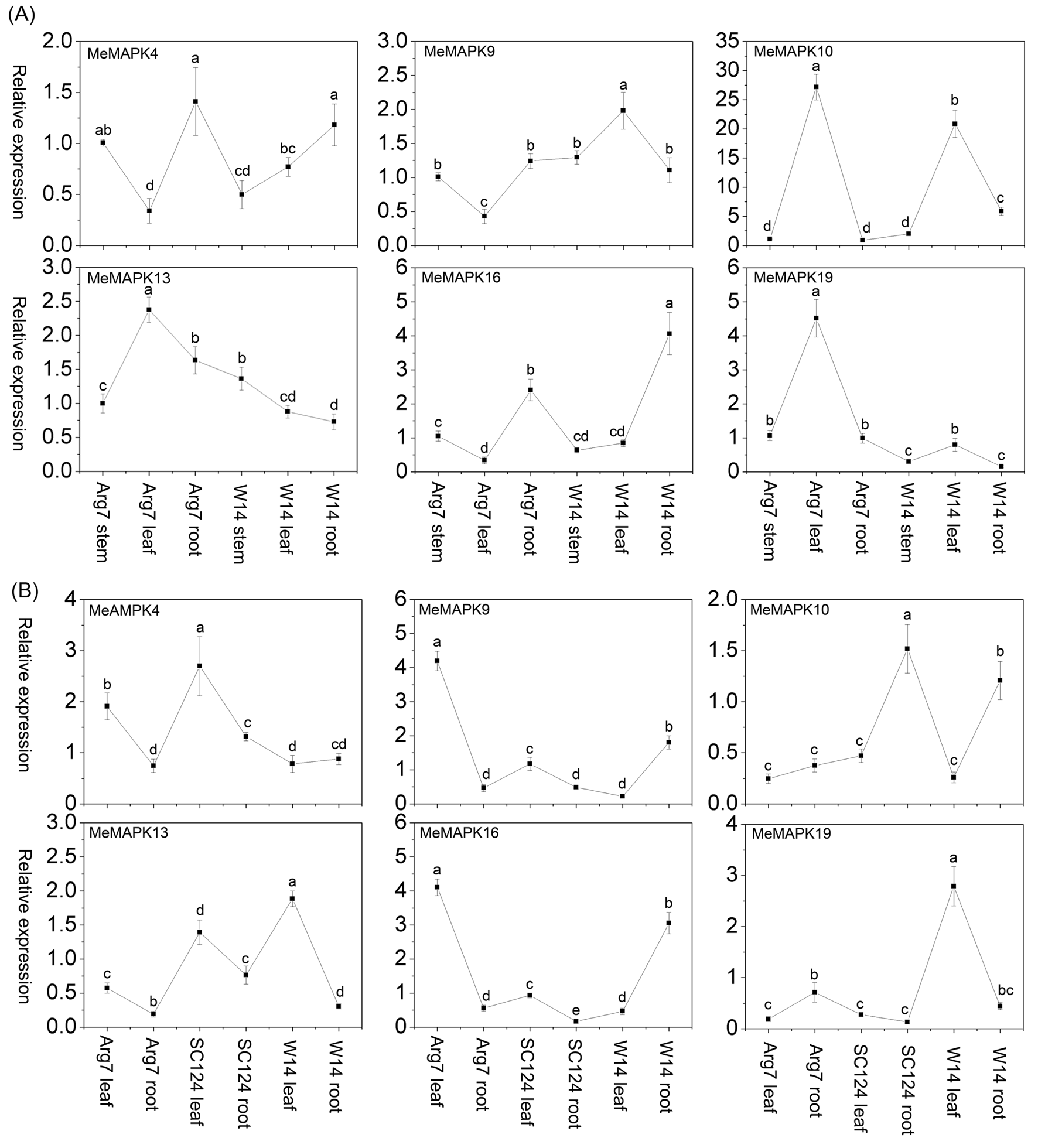

Supplement: Figure S11 — Relative expression levels of 6 MeMAPK genes in different tissues and in response to drought stress were examined by qRT-PCR analysis. (A) Expression profiles of MAPK4, −9, −10, −13, −16, and −19 in different tissues of Arg7 and W14. The mRNA fold difference was relative to that of Arg7 stem samples as calibrator; (B) expression profiles of MAPK4, −9, −10, −13, −16, and −19 in response to drought stress in Arg7, SC124, and W14. The mRNA fold difference was relative to that of untreated samples as calibrator. Data are means ± SE calculated from three biological replicates. Values with the same letter were not significantly different according to Duncan's multiple range tests (P < 0.05, n = 3). [file Image11.TIF]
